# NAT10 Promotes Gastric Cancer Liver Metastasis by Modulation of M2 Macrophage Polarization and Metastatic Tumor Cell Hepatic Adhesion

**DOI:** 10.1002/advs.202410263

**Published:** 2025-02-22

**Authors:** Chen Chen, Zhangding Wang, Qingfeng Lin, Mengmeng Li, Lei Xu, Yao Fu, Xiaoya Zhao, Zhuang Ma, Jiawen Xu, Shimeng Zhou, Mingyue Zhang, Yun Qian, Linsen Bao, Bo Wang, Meng Wang, Qingqing Ding, Qiang Wang, Shouyu Wang

**Affiliations:** ^1^ Department of Hepatobiliary Surgery, The First Affiliated Hospital of Anhui Medical University; MOE Innovation Center for Basic Research in Tumor Immunotherapy Anhui Province Key Laboratory of Tumor Immune Microenvironment and Immunotherapy Hefei 230022 China; ^2^ Department of Oncology Jiangyin Clinical College of Xuzhou Medical University Jiangyin Hospital Affiliated to Nantong University Jiangyin People's Hospital Jiangyin 214400 China; ^3^ Medical School of Nanjing University Nanjing 210093 China; ^4^ Department of Gastroenterology The Affiliated Drum Tower Hospital of Nanjing University Medical School Nanjing 210008 China; ^5^ Department of Pathology The First Affiliated Hospital of Anhui Medical University Hefei 230022 China; ^6^ Division of Gastric Surgery Department of General Surgery The Affiliated Drum Tower Hospital of Nanjing University Medical School Nanjing 210008 China; ^7^ Department of Geriatric Oncology The First Affiliated Hospital of Nanjing Medical University Nanjing 210029 China

**Keywords:** cell adhesion, gastric cancer, liver metastasis, macrophage polarization, NAT10

## Abstract

The relationship between patterns of RNA modifications and gastric cancer (GC) liver metastasis (GCLM) remains unclear. Here, by single‐cell sequencing, clinical sample analysis, and mouse model studies, an abnormal increase in the expression of the RNA acetyltransferase N‐acetyltransferase 10 (NAT10) in liver metastatic GC cells is identified. NAT10‐mediated N4‐acetylcytidine modification of CXCL2 and KLF5 mRNA increases their stability. Then, secreted CXCL2 is found to promote the infiltration and polarization of M2‐like macrophages to produce oncostatin M, which transcriptionally activates NAT10 expression via STAT3 signaling. In addition, organoid models confirm that NAT10 promotes the adhesion of GC cells to hepatocytes. Mechanistically, KLF5 transcriptionally activates ITGαV, facilitating GC cell attachment to hepatocytes. Intriguingly, high expression of NAT10/KLF5 axis is associated with poor prognosis of GC patients and targeting this axis significantly reduces GCLM in preclinical murine models. Collectively, these findings suggest the clinical significance of NAT10 in developing targeted therapies for GC patients with liver metastasis.

## Graphical abstract

The N‐acetyltransferase‐10 (NAT10)‐mediated mRNA N4‐acetylcytidine modification enhances the stability of CXCL2 and KLF5 mRNA. Then, secreted CXCL2 promotes M2 macrophage polarization and oncostatin M secretion, activating NAT10 expression via STAT3 signaling. Additionally, KLF5 upregulates ITGαV, facilitating gastric cancer cell attachment to hepatocytes, both contributing to gastric cancer liver metastasis.

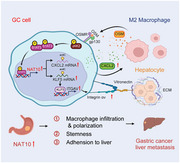

## 1 Introduction

Gastric cancer (GC) is the fifth most common cancer and the fourth leading cause of cancer‐related death worldwide.^[^ ^1^ ^]^ Despite recent advancements in the early detection and treatment of GC, a substantial number of patients are still diagnosed with GC only after it has progressed to an advanced stage, which is accompanied by the development of distant metastasis.^[^ ^2^ ^]^ The process of metastatic spread involves intricate interactions between malignant cells and the host, exhibiting organ selectivity.^[^ ^3^ ^]^ The liver is particularly common site of metastasis in advanced GC, and liver metastasis is associated with a significantly poorer prognosis for patients.^[^ ^4^ ^]^ Therefore, it is of utmost importance to gain a comprehensive understanding of the molecular mechanisms that drive liver metastasis in GC.

The tumor microenvironment (TME) is a complex network comprising cancer cells, blood vessels, immune cells, fibroblasts, cytokines, and extracellular matrix, all of which play pivotal roles in tumor progression.^[^ ^3^, ^5^ ^]^ Among the various components of the TME, tumor‐associated macrophages (TAMs), particularly immunosuppressive and protumorigenic M2‐like macrophages, are highly abundant in advanced tumors.^[^ ^3^, ^5^ ^]^ Previous studies have indicated that M2‐like macrophages promote tumor progression by releasing proliferative cytokines (such as EGF, IL‐23, and VEGF) that facilitate angiogenesis, lymphangiogenesis, and the growth of cancer cells. Moreover, M2‐like macrophages secrete anti‐inflammatory cytokines (such as CCL20) that promote the development of regulatory T cells.^[^ ^6^ ^]^ However, the current understanding of the mechanisms underlying the recruitment of M2‐like macrophages and the specific roles of these cells in the liver metastasis of GC is still incomplete.

Epigenetic modifications are heritable changes in genetic material that do not alter the nucleotide sequence, and they contribute to heritable phenotypic changes.^[^ ^7^ ^]^ Among epigenetic modifications, RNA modifications are essential for maintaining cell homeostasis and influencing the progression of various diseases by regulating gene expression.^[^ ^8^ ^]^ Over 170 modifications have been identified in RNA, with a particular emphasis on abundant classes of RNA such as rRNA and tRNA; among these RNA modifications, N6‐methyladenosine (m6A), N4‐acetylcytidine (ac4C), 5‐methylcytidine (m5C), N7‐methylguanosine (m7G), and N1‐methyladenosine (m1A) have gained increasing attention in terms of their relationship to cancer progression, and our research group has focused specifically on the functional mechanism of m6A modification in different types of cancer.^[^ ^9^ ^]^ N‐acetyltransferase 10 (NAT10), the sole RNA acetyltransferase catalyzing ac4C modification, has been implicated in various cancers through its functions in maintaining mRNA stability and increasing translation efficiency.^[^ ^10^ ^]^ However, the exact relationship between ac4C modification and liver metastasis in GC remains unknown.

In this study, we first investigated the expression patterns of 30 regulatory enzymes associated with five common RNA modifications (ac4C, m6A, m1A, m5C, and m7G) using single‐cell RNA sequencing (scRNA‐seq). Among these enzymes, NAT10 was significantly upregulated in liver metastatic GC cells. Further experiments revealed that NAT10 plays a crucial role in promoting the recruitment and polarization of M2‐like macrophages by increasing the stability of CXCL2 mRNA in an ac4C‐dependent manner in liver metastatic GC cells. Interestingly, M2‐like macrophages were found to secrete oncostatin M (OSM), which activated the STAT3 signaling pathway and further promoted NAT10 transcription in GC cells. Additionally, NAT10 was found to increase the ability of gastric cancer cells to adhere to hepatocytes via ac4C modification of KLF5 mRNA, leading to increased transcription of ITGαV. Notably, inhibition of the NAT10/KLF5 axis effectively suppressed liver metastasis in GC. Overall, our study suggests that NAT10 is a promising predictive biomarker and therapeutic target for GC with liver metastasis.

## 2 Results

### 2.1 NAT10‐Mediated ac4C Modification Is Increased in Liver Metastases of GC

**Figure 1:**
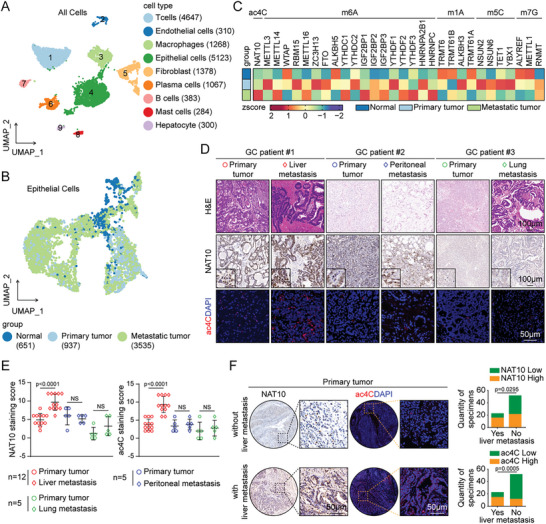
NAT10‐mediated ac4C modification is increased in liver metastases of GC. A) Uniform manifold approximation and projection (UMAP) plot showing the cell types of the 14 760 high‐quality cells (left panel), and a summary of the numbers of cells of each assigned type (right panel). B) UMAP embedding of the inferred CopyKAT diploid (normal cells) and aneuploid (primary or metastatic tumor cells) copy number profiles (all epithelial cells). C) Heatmap of 30 regulatory enzymes related to ac4C, m6A, m1A, m5C, and m7G in normal, primary tumor, and metastatic tumor cells after *z* score transformation. D) H&E staining (scale bars = 100 µm), IHC staining of NAT10 (scale bars = 100 µm), and IF staining of ac4C in primary tumors, liver metastases, peritoneal metastases, and lung metastases (scale bars = 50 µm). E) The distributions of the NAT10 and ac4C staining scores in primary tumors and the corresponding liver metastatic tumors (*n* = 12), peritoneal metastatic tumors (*n* = 5), and lung metastatic tumors (*n* = 5) were determined. F) Representative images of the GC TMA (cohort 1) subjected to IHC staining with the anti‐NAT10 antibody and to IF staining with the anti‐ac4C antibody (scale bars = 50 µm) are shown (left panel). The proportions of specimens showing a low or high NAT10 expression level and a low or high ac4C abundance relative to the incidence of liver metastasis are shown (right panel). GC, gastric cancer; UMAP, uniform manifold approximation and projection; IHC, immunohistochemical; IF, immunofluorescence. Data are represented as mean ± standard error of the mean (SEM) of three independent experiments. NS, not significant.

To explore the cellular and molecular heterogeneity in liver metastatic GC, two endoscopic biopsies of primary GC tissue and paired ultrasound‐guided puncture biopsies of liver metastatic tissues were subjected to single‐cell analysis (Figure S1A, Supporting Information). After quality control and cell filtering, 14 760 cells were retained for further analysis. Then, graph‐based clustering was performed, resulting in the identification of 21 clusters after normalizing gene expression and conducting principal component analysis (Figure S1B,C, Supporting Information). These clusters were characterized as T cells (4647 cells, expressing PTPRC and CD3D), endothelial cells (310 cells, expressing PECAM1 and ENG), macrophages (1268 cells, expressing CD68 and CD14), epithelial cells (5123 cells, expressing EPCAM and CD24), fibroblasts (1378 cells, expressing ACTA2 and DCN), plasma cells (1067 cells, expressing MZB1 and SDC1), B cells (383 cells, expressing CD79A and MS4A1), mast cells (284 cells, expressing TPSAB1 and TPSB2), and hepatocytes (300 cells, expressing ALB and ALDOB) (**Figure** **1**A and S1D (Supporting Information)). Moreover, these cells were assigned to primary tumors (5297 cells) or liver metastases (9463 cells) (Figure S1E, Supporting Information).

In parallel, we analyzed copy number variations in epithelial cells using the CopyKAT algorithm, and the results showed that there were 651 normal cells, 937 primary tumor cells, and 3535 metastatic tumor cells (Figure 1B). Subsequently, to further explore the role of RNA modification in the liver metastasis of GC, the expression patterns of 30 regulatory enzymes associated with five common RNA modifications, namely, ac4C, m6A, m1A, m5C, and m7G, were further examined at the single‐cell level. Intriguingly, NAT10, the core acetyltransferase for ac4C, and m6A‐related methyltransferases such as METTL14 were significantly upregulated in liver metastases of patients with GC, while the other enzymes were not significantly differentially expressed (Figure 1C).

Consistently, we further confirmed that the protein expression of NAT10 and the abundance of ac4C were significantly greater in liver metastatic tissues than in the paired primary tumor tissues from GC patients; however, no significant differences were found in the lung or peritoneal metastases (Figure 1D,E). Moreover, in a GC patient with metastases in multiple organs, the liver metastases showed concurrent increases in NAT10 expression and the ac4C abundance compared to the peritoneal metastases (Figure S1F, Supporting Information). To further explore the association between NAT10 expression and liver metastasis in GC, we performed immunohistochemical (IHC) staining on a tissue microarray (TMA) containing 75 primary tumor samples (cohort 1) from GC patients with liver metastasis (*n* = 23) or without liver metastasis (*n* = 52). The results revealed that the primary GC tissues from the patients with liver metastasis exhibited higher NAT10 expression (Figure 1F). Similarly, a higher level of ac4C modification was also positively correlated with liver metastasis (Figure 1F). Taken together, these data suggest that abnormal increases in NAT10 expression and the ac4C abundance in GC cells may indicate an increased risk for liver metastasis.

### 2.2 NAT10 Facilitates the Liver Metastasis of GC In Vivo

**Figure 2:**
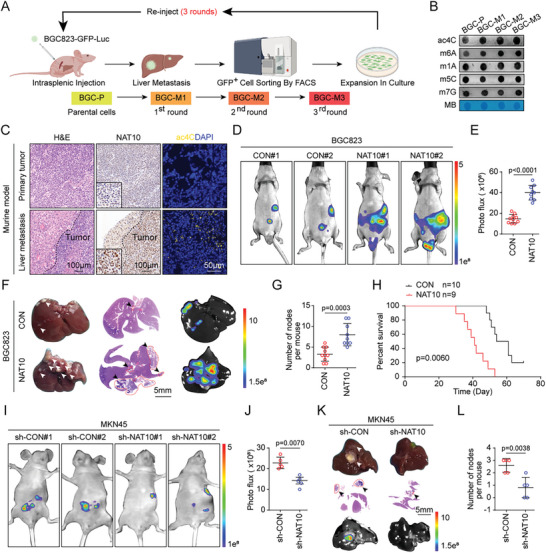
NAT10 facilitates the liver metastasis of GC in vivo. A) Generation of liver metastatic cells through intrasplenic reinjection. B) mRNA isolated from BGC‐P, BGC‐M1, BGC‐M2, and BGC‐M3 cells was subjected to dot blot analyses with anti‐ac4C, anti‐m6A, anti‐m1A, anti‐m5C, and anti‐m7G antibodies, and MB was used as the loading control. C) H&E staining (scale bars = 100 µm), IHC staining of NAT10 (scale bars = 100 µm), and IF staining of ac4C (scale bars = 50 µm) in primary tumors and liver metastases from the mouse model. D–H) Overexpression of NAT10 significantly increased GCLM in nude mice (*n* = 5). (D) Representative bioluminescence images of mice 4 weeks after intrasplenic injection of BGC823 cells with NAT10 overexpression or the corresponding control cells and (E) quantification of the bioluminescence intensity in the liver region. (F) Representative images of the metastatic nodules in the liver (left panel, bright field; middle panel, H&E staining (scale bars = 5 mm); right panel, bioluminescence imaging) and (G) quantification of the metastatic nodules. Arrow: liver metastasis. (H) Kaplan–Meier survival curves of nude mice with liver metastases resulting from intrasplenic injection of BGC823 cells overexpressing NAT10 or the corresponding control cells (BGC‐CON, *n* = 10; BGC‐NAT10, *n* = 9). I–L) NAT10 knockdown significantly decreased GCLM in nude mice (*n* = 5). (I) Representative bioluminescence images of mice 4 weeks after intrasplenic injection of MKN45 cells with NAT10 knockdown or the corresponding control cells and (J) quantification of the bioluminescence intensity in the liver region. (K) Representative images of the metastatic nodules in the liver (upper panel, bright field; middle panel, H&E staining (scale bars = 5 mm); lower panel, bioluminescence imaging) and (L) quantification of the metastatic nodules. Arrow: liver metastasis. OE, overexpressing; KD, knockdown. Data are represented as mean ± SEM of three independent experiments. NS, not significant.

To further confirm the role of RNA modification in GC liver metastasis (GCLM), we first established isogenic liver metastatic GC cell lines by three rounds of selection of liver metastatic subpopulations in vivo using BGC823‐GFP‐Luc parental cells (BGC‐P) and designated the cells established in the sequential rounds of selection BGC‐M1, BGC‐M2, and BGC‐M3 (**Figure** **2**A). The dot blot assay showed that the abundances of ac4C and m6A on total mRNA were significantly greater in liver metastatic cells (BGC‐M1, BGC‐M2, and BGC‐M3) than in BGC‐P cells (Figure 2B). Furthermore, we observed the expected increases in the expression of NAT10 and the abundance of ac4C in liver metastatic lesions compared to primary tumors (Figure 2C). To further clarify the function of NAT10 in GCLM, we constructed GC cell lines (BGC823 and MGC803) with stable NAT10 overexpression (Figure S2A,B, Supporting Information). Subsequently, we injected BGC823‐Luc cells overexpressing NAT10, along with control cells, into nude mice through the splenic vein. Four weeks later, we found that NAT10 upregulation significantly promoted GCLM, as shown by bioluminescence imaging (Figure 2D,E), and increased the number and size of liver metastatic lesions compared to those in the control groups (Figure 2F,G). Notably, NAT10 overexpression also resulted in a decrease in mouse survival (Figure 2H). In addition, we constructed GC cell lines (AGS and MKN45) with stable NAT10 knockout or knockdown (Figure S2C–E, Supporting Information). As expected, NAT10 deficiency significantly suppressed the liver metastasis of these cells (Figure 2I–L). Overall, these findings underscore the critical role of NAT10 in promoting the liver metastasis of GC.

### 2.3 NAT10 Recruits and Polarizes M2‐Like Macrophages via ac4C Modification of CXCL2 mRNA

**Figure 3:**
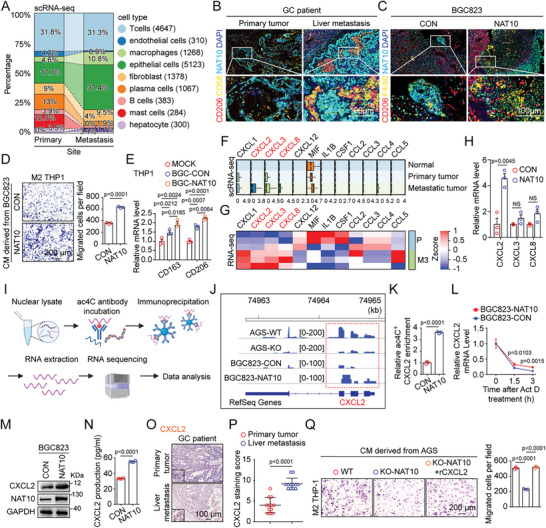
NAT10 recruits and polarizes M2‐like macrophages via ac4C modification of CXCL2 mRNA. A) scRNA‐seq data showing the proportions of each cell type in the normal, primary tumor, and metastatic tumor groups. B) MIF staining showing the differences in the expression of NAT10, the number of macrophages (marked by CD68), and the number of M2‐like macrophages (marked by CD206) between the primary tumors and liver metastases of GC patients. C) MIF staining showing the differences in the expression of NAT10, the number of macrophages (marked by F4/80), and the number of M2‐like macrophages (marked by CD206) between the liver metastases of nude mice injected intrasplenically with BGC823 cells overexpressing NAT10 or the corresponding control cells. D) M2‐like macrophages derived from THP‐1 cells that migrated through uncoated filter membranes after 16 h of culture in medium derived from BGC823 cells with NAT10 overexpression or the corresponding control cells. The cells were stained with crystal violet, visualized via microscopy (left panel, scale bars = 200 µm), and counted (right panel). E) qPCR was conducted to measure the mRNA levels of M2‐like markers (CD163 and CD206) in THP‐1 cells cultured with medium derived from BGC823 cells with NAT10 overexpression or the corresponding control cells. F,G) CXCL2, CXCL3, and CXCL8 were identified as overlapping genes between the scRNA‐seq and RNA‐seq (BGC‐P vs BGC‐M3 cells) data. (F) Box plots showing differences in the expression of well‐known macrophage recruitment‐related chemokines among normal, primary tumor, and metastatic tumor cells, as determined via scRNA‐seq. (G) Heatmap showing differences in well‐known macrophage recruitment‐related chemokines between BGC‐P and BGC‐M3 cells according to RNA‐seq. H) The mRNA levels of CXCL2, CXCL3, and CXCL8 in NAT10‐OE GC cells were measured by qRT‐PCR. I) Schematic overview of the experimental design for acRIP‐seq based on cells with stable overexpression and knockout of NAT10 compared with their corresponding controls. J) The ac4C abundance on CXCL2 mRNA transcripts in BGC823 cells and AGS cells, as determined by acRIP‐seq. K) acRIP–qPCR analysis was employed to demonstrate NAT10‐mediated CXCL2 ac4C modification. ac4C modification of CXCL2 increased upon overexpression of NAT10. L) The levels of CXCL2 expression in NAT10‐OE and the corresponding control GC cells treated with actinomycin D (2 µg mL^−1^) at the indicated time points were measured by qRT‐PCR. M) The protein level of CXCL2 in NAT10‐OE GC cells was measured by western blotting. N) The concentration of CXCL2 in the culture supernatant of NAT10‐OE GC cells was measured by ELISA. O) The protein level of CXCL2 in primary tumor and liver metastatic tumor tissues from GC patients was evaluated by IHC staining (scale bars = 100 µm). P) The distribution of the CXCL2 staining score in primary tumors and the corresponding liver metastatic tumors (*n* = 12) was determined. Q) Representative images (left panel) and quantification (right panel) of migrated M2‐like macrophages derived from THP‐1 cells cultured in medium derived from NAT10‐KO AGS cells supplemented with human rCXCL2. The cells were stained with crystal violet and visualized via microscopy (scale bar = 200 µm). GC, gastric cancer; scRNA‐seq, single‐cell RNA sequencing; RNA‐seq, RNA sequencing; IHC, immunohistochemical; MIF, multiplex immunofluorescence; OE, overexpressing; KO, knockout. Data are represented as mean ± SEM of three independent experiments. NS, not significant.

To elucidate the differences in the tumor microenvironment between primary GC tissues and liver metastatic lesions, the distribution of the cell lineages was further analyzed. Intriguingly, the proportions of epithelial cells and macrophages in metastatic tissues were dramatically increased, but those of plasma cells, B cells, and mast cells were significantly decreased (**Figure** **3**A). Furthermore, multiplex immunofluorescence (MIF) staining confirmed the increased abundance of macrophages, particularly M2‐like macrophages, in liver metastatic lesions compared to the paired primary GC tumors (Figure 3B). Furthermore, upregulation of NAT10 in GC cells significantly facilitated the recruitment of M2‐like macrophages to the liver metastatic niche, as demonstrated by MIF staining in a nude mouse model (Figure 3C). Moreover, treatment with conditioned medium (CM) from BGC823 and MGC803 cells overexpressing NAT10 increased the recruitment of M2‐like macrophages derived from THP‐1 cells in vitro (Figure 3D and Figure S3A (Supporting Information)). Simultaneously, treatment with this CM resulted in the polarization of more M0 THP‐1 cells to M2‐like phenotype than did treatment with CM from the corresponding control cells (Figure 3E and Figure S3B (Supporting Information)). By contrast, knockout of NAT10 had the opposite effects in vitro (Figure S3C,D, Supporting Information).

Next, we hypothesized that the specific chemokines secreted by metastatic tumor cells might play a crucial role in the recruitment and polarization of M2‐like macrophages. Interestingly, the scRNA‐seq data revealed the upregulation of CXCL2, CXCL3, CXCL8, and CCL4 in metastatic tumor cells compared to primary tumor cells or normal cells (Figure 3F). RNA‐seq data from BGC‐P and BGC‐M3 cells further confirmed the elevated levels of CXCL1, CXCL2, CXCL3, and CXCL8 in BGC‐M3 cells compared to BGC‐P cells (Figure 3G). Analysis of three transcripts that overlapped between the scRNA‐seq data and the RNA‐seq data revealed that only CXCL2 was consistently regulated by NAT10 in both the NAT10‐overexpressing BGC823 cells and the NAT10‐knockout AGS cells (Figure 3H and Figure S3E (Supporting Information)). It has been reported that NAT10 can increase the stability or translation efficiency of mRNA.^[^ ^10^, ^11^ ^]^ We thus performed ac4C‐modified RNA immunoprecipitation sequencing (acRIP‐seq) in GC cells with stable overexpression of NAT10 or stable knockout of NAT10 and the corresponding control cells. The results indicated a significant increase in the ac4C abundance in CXCL2 mRNA upon NAT10 overexpression and a decrease upon NAT10 knockout (Figure 3I,J), which were further verified by acRIP–quantitative polymerase chain reaction (qPCR) in NAT10‐overexpressing or knockout GC cells (Figure 3K and Figure S3F (Supporting Information)). Furthermore, a RNA decay assay revealed that overexpression of NAT10 preserved the stability of CXCL2 mRNA, whereas NAT10 knockout led to its instability (Figure 3L and Figure S3G (Supporting Information)). We also confirmed that overexpression of NAT10 increased the CXCL2 protein level, while knockout of NAT10 or treatment with Remodelin, an inhibitor of NAT10,^[^ ^12^ ^]^ reduced the CXCL2 protein level (Figure 3M and Figure S3H (Supporting Information)). Consistent with these results, the secretion of CXCL2 into the CM was increased in GC cells overexpressing NAT10 (Figure 3N). As expected, we also found that the secretion of CXCL2 into the CM was greater in BGC‐M3 cells than in BGC‐P cells (Figure S3I, Supporting Information).

Intriguingly, we found that the expression of CXCL2 was greater in the liver metastatic niche than in the primary tumor in GC patients (Figure 3O,P) and that it was also upregulated in the metastatic lesions in the liver compared to those in the peritoneum in GC patient with multiorgan metastases, as confirmed by IHC staining (Figure S3J, Supporting Information). Moreover, IHC staining demonstrated that NAT10 overexpression dramatically increased CXCL2 staining in the liver metastasis mouse model (Figure S3K, Supporting Information). Moreover, treatment with recombinant CXCL2 protein (rCXCL2) reversed the NAT10‐knockout‐induced decrease in the recruitment of M2‐like macrophages derived from THP‐1 cells by AGS cells (Figure 3Q). Collectively, our data demonstrated that NAT10 promoted the liver metastasis of GC through CXCL2‐mediated M2‐like macrophage recruitment and polarization.

### 2.4 M2‐Like Macrophage‐Secreted OSM Activates NAT10 Transcription in Liver Metastatic GC via STAT3 Signaling

**Figure 4:**
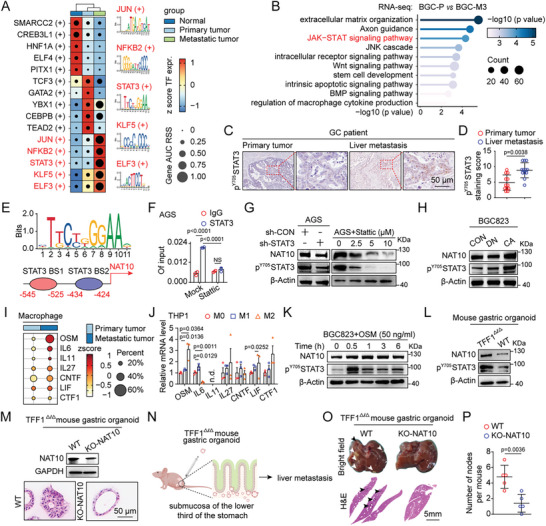
M2‐like macrophage‐secreted OSM activates NAT10 transcription in liver metastatic GC via STAT3 signaling. A) Heatmap and dot plot showing TF expression levels on a color scale and gene AUC RSSs on a size scale. Cell groups are ordered on the basis of their gene expression and annotated as normal, primary tumor, or metastatic tumor cells. The top five TFs in metastatic tumor cells, as determined by SCENIC+, were JUN, NFKB2, STAT3, KLF5, and ELF3. B) KEGG enrichment analysis results showing that the JAK–STAT signaling pathway was enriched in genes expressed in BGC‐M3 cells. C) The level of p^Y705^ STAT3 in primary tumor and liver metastatic tumor tissues from GC patients was evaluated by IHC staining (scale bars = 50 µm). D) The distribution of the p^Y705^ STAT3 staining score in primary tumors and the corresponding liver metastatic tumors (*n* = 12) was determined. E) Enriched sequences among STAT3–DNA cross‐linking sites and STAT3 binding sites in the promoter region of NAT10 were predicted by the JASPAR database. F) Enrichment of STAT3 at the NAT10 promoter, as determined by ChIP–qPCR, upon STAT3 inhibitor treatment. G,H) The protein level of NAT10 in (G) STAT3‐KD AGS cells and AGS cells treated with a STAT3 inhibitor, (H) cells transfected with plasmids encoding the constitutively active STAT3 mutant (STAT3‐CA) was measured by western blotting. I) Dot plot of genes encoding cytokines related to the activation of STAT3 signaling mapped to macrophages in primary tumors and metastatic tumors. J) qRT‐PCR was performed to measure the mRNA levels of cytokines in polarized THP‐1 cells. K) The protein levels of p^Y705^ STAT3 and NAT10 in BGC823 cells treated with OSM (50 ng mL^−1^) were measured by western blotting. L) The protein levels of p^Y705^ STAT3 and NAT10 in mouse gastric organoids (WT, TFF1^∆/∆^) were measured by western blotting. M) The protein levels of p^Y705^ STAT3 and NAT10 in mouse gastric organoids (TFF1^∆/∆^‐WT, TFF1^∆/∆^‐NAT10‐KO) were measured by western blotting and H&E staining (scale bars = 50 µm). N–P) Knockout of NAT10 in TFF1^∆/∆^ organoids significantly decreased GCLM in nude mice (*n* = 5). (N) Schematic overview of the experimental design showing organoid injection into the submucosa of the lower third of the stomach in nude mice. (O) Representative images of the metastatic nodules in the liver (upper panel, bright field; lower panel, H&E staining (scale bars = 5 mm). Arrow: liver metastasis. (P) Quantification of the metastatic nodules. SCENIC+, single‐cell multiomic inference of enhancers and gene regulatory networks; KEGG, Kyoto Encyclopedia of Genes and Genomes; GC, gastric cancer; ChIP, chromatin immunoprecipitation; KD, knockdown; KO, knockout; CA, constitutively active. Data are represented as mean ± SEM of three independent experiments. NS, not significant.

To further explore the upstream regulatory mechanism underlying the high NAT10 expression in liver metastatic GC, we then employed single‐cell multiomic inference of enhancers and gene regulatory networks (SCENIC+) to identify the transcription factors (TFs) maintaining the high expression of NAT10 in liver metastatic GC. SCENIC+ identified several master regulators in metastatic GC cells, with the top five TFs being JUN, NFKB2, STAT3, KLF5, and ELF3 (**Figure** **4**A). Kyoto Encyclopedia of Genes and Genomes (KEGG) pathway analysis of the RNA‐seq data (BGC‐P vs BGC‐M3 cells) revealed enrichment in extracellular matrix organization, axon guidance and the JAK–STAT signaling pathway in BGC‐M3 cells (Figure 4B). Consistently, the JAK–STAT signaling pathway was also identified as enriched by analysis of the scRNA‐seq data (Figure S4A, Supporting Information). In addition, activation of STAT3 was observed in liver metastases compared to the paired primary tumors, as indicated by the level of phosphorylated STAT3 (p‐STAT3, Y705) (Figure 4C,D). Moreover, two potential STAT3 binding sites were identified in the NAT10 promoter region (Figure 4E). Chromatin immunoprecipitation (ChIP) assays further demonstrated the binding of STAT3 to the NAT10 promoter, and this binding ability was reduced when STAT3 signaling was blocked by treatment with the STAT3‐specific inhibitor Stattic (Figure 4F). Furthermore, the mRNA and protein levels of NAT10 were decreased upon depletion of p‐STAT3 using shRNA and a STAT3 inhibitor (Figure 4G and Figure S4B,C (Supporting Information)). By contrast, a constitutively active STAT3 mutant (STAT3‐CA), but not a dominant‐negative STAT3 mutant (STAT3‐DN), could significantly increase the expression of NAT10 (Figure 4H).

Subsequently, we hypothesized that M2‐like macrophages might be involved in STAT3 activation and the subsequent increase in NAT10 expression. Our scRNA‐seq analysis revealed that OSM was the most upregulated cytokine related to JAK/STAT3 signaling activation (Figure 4I). Furthermore, we found that M2‐like THP‐1 cells exhibited a greater increase in the expression of OSM than M0‐ or M1‐like THP‐1 cells (Figure 4J). Moreover, ChIP assays demonstrated that STAT3 was enriched at the promoter region of NAT10 when BGC823 cells were stimulated with 50 ng mL^−1^ OSM (Figure S4D, Supporting Information). Similarly, western blot analysis further confirmed that OSM triggered the phosphorylation of STAT3 and increased the protein level of NAT10 in BGC823 cells (Figure 4K).

It has been reported that loss of TFF1 expression in TFF1‐knockout (KO) (TFF1^∆/∆^) mice promotes STAT3 activation and leads to a proinflammatory phenotype that includes spontaneous development of adenocarcinomas.^[^ ^13^ ^]^ We then generated gastric organoids from 20 months old TFF1^∆/∆^ mice and the counterpart wild type (WT) mice and found that the organoids from TFF1^∆/∆^ mice had significantly increased protein levels of p‐STAT3 and NAT10 (Figure 4L). Moreover, stable NAT10‐KO organoids were established using TFF1^∆/∆^ organoids and then injected into the submucosa of the lower third of the stomach in nude mice (Figure 4M,N). After 2 months, recipient mice injected with TFF1^∆/∆^‐NAT10‐KO organoids had fewer liver metastatic nodules than those injected with TFF1^∆/∆^‐WT organoids (Figure 4O,P). Taken together, these results provide compelling evidence indicating that M2‐like macrophages in liver metastatic GC secrete large quantities of OSM to activate STAT3 signaling and in turn promote NAT10 transcription.

### 2.5 NAT10 Facilitates the Adhesion of GC Cells to Hepatocytes by Upregulating KLF5

**Figure 5:**
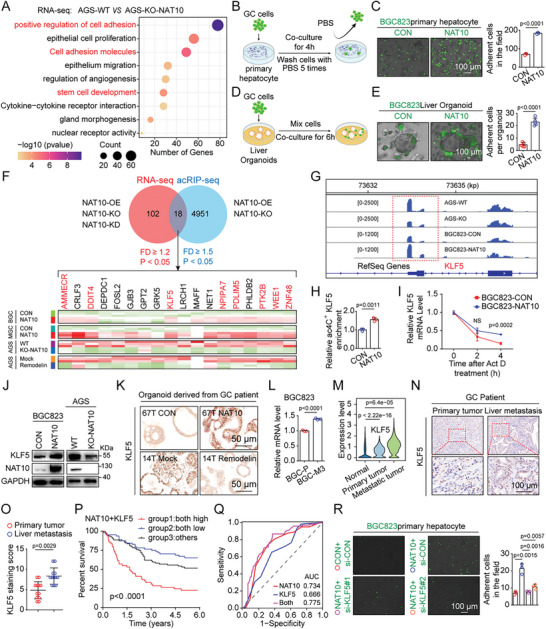
NAT10 facilitates the adhesion of GC cells to hepatocytes by upregulating KLF5. A) GO enrichment analysis of RNA‐seq data (AGS‐WT vs AGS‐KO‐NAT10) showing the enrichment of genes involved in the positive regulation of cell adhesion, cell adhesion molecules, and stem cell development. B,C) Overexpression of NAT10 significantly promoted the adhesion of GC cells to hepatocytes. (B) Schematic overview of the experimental design showing GC cells adhering to hepatocytes. (C) Representative images of NAT10‐OE BGC823 cells adhering to hepatocytes (left panel, scale bars = 100 µm) and quantification of the adherent cells (right panel). D,E) Overexpression of NAT10 significantly promoted the adhesion of GC cells to normal human liver organoids. (D) Schematic overview of the experimental design showing GC cells adhering to normal human liver organoids. (E) Representative images of NAT10‐OE BGC823 cells adhering to normal human liver organoids (left panel, scale bars = 100 µm) and quantification of the adherent cells (right panel). F) RNA‐seq and acRIP‐seq identified differentially expressed genes in stable NAT10‐OE, stable NAT10‐KO, and stable NAT10‐KD cells compared with their corresponding controls, and 18 transcripts were identified as overlapping between the two datasets (upper panel). Heatmap showing the mRNA levels of the transcripts in NAT10‐OE, NAT10‐KO, and Remodelin‐treated GC cells measured by qRT‐PCR (lower panel). G) The ac4C abundance on KLF5 mRNA transcripts in BGC823 cells and AGS cells, as determined by acRIP‐seq. H) acRIP–qPCR analysis was employed to demonstrate NAT10‐mediated KLF5 ac4C modification. ac4C modification of KLF5 increased upon overexpression of NAT10. I) The level of KLF5 expression in NAT10‐OE and the corresponding control GC cells treated with actinomycin D (2 µg mL^−1^) at the indicated time points was measured by qRT‐PCR. J) The protein level of KLF5 in NAT10‐OE and NAT10‐KO GC cells was measured by western blotting. K) The level of KLF5 in NAT10‐OE and Remodelin‐treated human GC organoids was evaluated by IHC staining (scale bars = 50 µm). L) The level of KLF5 expression in BGC‐P and BGC‐M3 cells was measured by qRT‐PCR. M) Violin plot showing the differences in the expression of KLF5 among normal, primary tumor, and metastatic tumor cells, as determined via scRNA‐seq. N) The protein level of KLF5 in primary tumor and liver metastatic tumor tissues from GC patients was evaluated by IHC staining (scale bars = 100 µm). O) The distribution of the KLF5 staining score in primary tumors and the corresponding liver metastatic tumors (*n* = 12) was determined. P) GC patients (cohort 2) were divided into three subgroups according to the median expression value of each gene: high expression of both NAT10 and KLF5, low expression of both NAT10 and KLF5, and other expression patterns. Kaplan–Meier analysis of the three subgroups of GC patients. Q) ROC curve for NAT10 expression alone, KLF5 expression alone, and the combination of NAT10 expression and KLF5 expression in cohort 2. R) Representative images (left panel, scale bars = 100 µm) and quantification (right panel) of NAT10‐OE BGC823 cells transfected with si‐KLF5 adhering to hepatocytes. GC, gastric cancer; WT, wild type; KO, knockout; OE, overexpressing; IHC, immunohistochemical. Data are represented as mean ± SEM of three independent experiments. NS, not significant.

To elucidate the mechanism by which NAT10 promotes the liver metastasis of GC cells, Gene Ontology (GO) analysis of RNA‐seq data (AGS‐WT vs AGS‐KO‐NAT10 cells) revealed that cell‐adhesion‐related pathways were most significantly enriched in the differentially expressed genes (**Figure** **5**A), indicating that metastatic GC cells with high expression of NAT10 may have strong hepatic adhesion ability. Thus, GC cells were seeded on a layer of primary cultured hepatocytes, incubated for 4 h, and then washed, the results indicated that BGC823 cells overexpressing NAT10 exhibited increased adherence to the hepatocytes, while NAT10 knockout cells exhibited decreased adherence (Figure 5B,C and Figure S5A,B (Supporting Information)). However, there was no discernible differences in adhesion to human bronchial epithelial (HBE) cells (Figure S5C–F, Supporting Information). Consistently, NAT10‐overexpressing BGC823 cells exhibited increased adhesion to normal human liver organoids (Figure 5D,E and Figure S5G,H (Supporting Information)). Collectively, these data suggest that the increased adhesive ability of GC cells with high NAT10 expression may contribute to liver metastasis.

To further explore the underlying regulatory mechanism of NAT10, differential expression in the RNA‐seq data (fold change ≥ 1.2; *p* < 0.05) and acRIP‐seq data (fold change ≥ 1.5; *p* < 0.05) was comprehensively analyzed. The analysis identified 18 overlapping genes, which were further validated via quantitative real‐time reverse transcriptase‐PCR (qRT‐PCR), and we found that the expression patterns of eight genes were consistent with the sequencing results (Figure 5F). Further visualization with Integrative Genomics Viewer of these genes demonstrated that the abundance of ac4C on KLF5 mRNA exhibited a highly significant difference not only in NAT10‐overexpressing GC cells (*p* = 0.002) but also in NAT10‐knockout GC cells (*p* = 1.193E‐12) (Figure 5G and Figure S6A–G (Supporting Information)). Furthermore, acRIP–qPCR assays confirmed a significant increase in ac4C modification on KLF5 mRNA in NAT10‐overexpressing BGC823 cells and a decrease in NAT10‐knockout AGS cells (Figure 5H and Figure S7A (Supporting Information)). Subsequently, RNA decay assays demonstrated that NAT10 overexpression increased KLF5 mRNA stability, whereas NAT10 deficiency decreased KLF5 mRNA stability (Figure 5I and Figure S7B (Supporting Information)). Moreover, western blot analysis confirmed that the KLF5 protein level was positively regulated by NAT10 in different GC cell lines, while mutation of NAT10 in the RNA helicase domain (K290A) or the functional acetyltransferase domain (G641E) did not affect the KLF5 protein level (Figure 5J and Figure S7C,D (Supporting Information)). Furthermore, organoids derived from GC patients were used to demonstrate the relationship between NAT10 and KLF5, and the expression of KLF5 was found to be augmented by NAT10 overexpression and inhibited by the NAT10 inhibitor Remodelin (Figure 5K). Moreover, we observed that the expression of KLF5 was greater in BGC823‐M3 cells than in BGC823‐P cells (Figure 5L). Moreover, our scRNA‐seq analysis revealed a significant increase in KLF5 expression in liver metastatic tumor cells (Figure 5M). Accordingly, immunostaining for KLF5 in GCLM specimens from both humans and mice confirmed the increase in KLF5 expression (Figure 5N,O and Figure S7E–G (Supporting Information)). To further analyze the relationships between NAT10/KLF5 expression and the prognosis of GC patients, IHC staining was performed on a TMA containing 192 GC samples (cohort 2), and the results revealed that the group with high expression of both NAT10 and KLF5 had a significantly worse prognosis than the group with low expression of NAT10 and/or KLF5 (*p* < 0.001) (Figure 5P); this finding was further confirmed by univariate Cox regression analysis (hazard ratios (HR) = 1.332; 95% confidence interval (CI) (1.188–1.493); *p* = 9.432e‐07) (Figure S7H, Supporting Information). The area under the curve (AUC) also demonstrated that the combination of NAT10 and KLF5 had better predictive validity than either parameter alone (Figure 5Q).

Additionally, the sphere formation assay indicated that stable NAT10 overexpression significantly increased the sphere‐forming ability of GC cells, while NAT10 knockout decreased this ability, indicating that NAT10 could maintain the stemness of GC cells (Figure S8A–C, Supporting Information).

To further investigate whether the ability of NAT10 to promote GC cell adhesion depends on KLF5 expression, stable KLF5‐overexpressing (OE) and KLF5‐KO GC cells were generated, and cell adhesion assays revealed that KLF5 increased the adhesion ability of GC cells (Figure S9A–F, Supporting Information). However, the recruitment of M2‐like macrophages derived from THP‐1 cells was not regulated by KLF5 (Figure S9G, Supporting Information). Notably, NAT10‐induced cell stemness maintenance and adhesion were markedly suppressed when KLF5 expression was knocked down in NAT10‐overexpressing BGC823 cells (Figure 5R and Figure S10A (Supporting Information)). Correspondingly, the reductions in the cell adhesion ability and stemness resulting from NAT10 loss were reversed via overexpression of KLF5 (Figure S10B,C, Supporting Information). In conclusion, our findings support the idea that NAT10 promotes GC cell liver metastasis by increasing cell adhesion and maintaining stemness through the upregulation of KLF5.

### 2.6 NAT10‐Mediated ac4C Modification of KLF5 Activates ITGαV Transcription

**Figure 6:**
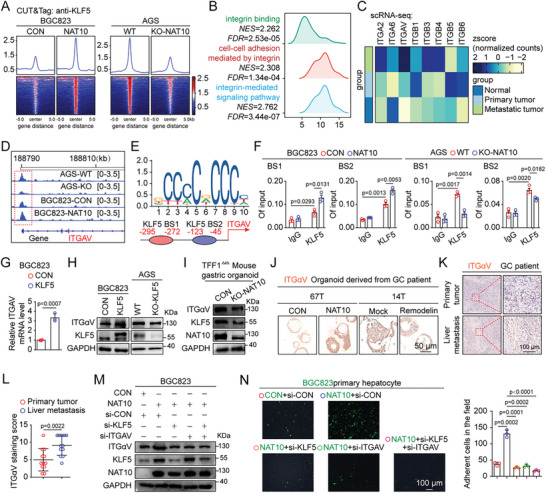
NAT10‐mediated ac4C modification of KLF5 mRNA and then activates ITGαV transcription. A) Density profiles (top) and heatmaps (bottom) of CUT&Tag‐seq data showing the read density in a ±5 kb window surrounding the center of the KLF5 target TSS in NAT10‐OE, NAT10‐KO, and the corresponding control cells. B) GSVA of CUT&Tag data showing that the integrin binding, cell–cell adhesion mediated by integrin, and integrin‐mediated signaling pathway gene sets were significantly enriched. C) Heatmap of integrin family genes related to metastasis in normal, primary tumor, and metastatic tumor cells, visualized as row *z* scores of the mean AUC values determined from scRNA‐seq data. D) IGV visualization of KLF5 occupancy in the promoter region of ITGAV. E) Enriched sequences among KLF5–DNA cross‐linking sites and KLF5 binding sites (BS1 and BS2) in the promoter region of ITGAV predicted by the JASPAR database. F) Enrichment of KLF5 at the ITGAV promoter, as determined by ChIP–qPCR, in NAT10‐OE and NAT10‐KO cells. G,H) The mRNA and protein levels of ITGAV (encoding ITGαV) in KLF5‐OE BGC823 cells and KLF5‐KO AGS cells were measured by qRT‐PCR and western blotting. I) The protein level of ITGαV in TFF1^∆/∆^‐WT and TFF1^∆/∆^‐NAT10‐KO organoids was measured by western blotting. J) The protein level of ITGαV in NAT10‐OE and Remodelin‐treated human GC organoids was evaluated by IHC staining (scale bars = 50 µm). K) The protein level of ITGαV in primary tumor and liver metastatic tumor tissues from GC patients was evaluated by IHC staining (scale bars = 100 µm). L) The distribution of the ITGαV staining score in primary tumors and the corresponding liver metastatic tumors (*n* = 12) was determined. M,N) Knockdown of KLF5/ITGαV axis suppressed the increase in cell adhesion caused by NAT10. (M) The protein expression levels of KLF5/ITGαV were measured in NAT10‐OE BGC823 cells treated with indicated siRNAs by western blotting. (N) Representative images (left panel, scale bars = 100 µm) and quantification (right panel) of indicated BGC823 cells that adhered to hepatocytes. CUT&Tag, cleavage under targets and tagmentation; GC, gastric cancer; OE, overexpressing; KO, knockout; AUC, area under the curve; IGV, Integrative Genomics Viewer; IHC, immunohistochemical. Data are represented as mean ± SEM of three independent experiments. NS, not significant.

Given that KLF5 functions as a classical transcription factor,^[^ ^14^ ^]^ we further evaluated whether NAT10 promotes the adhesion of GC cells by transcriptionally activating downstream genes through KLF5. To this end, we performed high‐throughput cleavage under targets and tagmentation (CUT&Tag) sequencing, and the data revealed a significant global increase in KLF5 occupancy upon NAT10 overexpression, while KLF5 occupancy was reduced upon NAT10 knockout (**Figure** **6**A). Intriguingly, gene set variation analysis (GSVA) revealed a positive correlation between KLF5 occupancy and the enrichment of gene sets related to integrin, such as integrin binding, cell–cell adhesion mediated by integrin, and integrin‐mediated signaling pathway (Figure 6B). Moreover, our scRNA‐seq analysis identified ITGAV as the most upregulated gene (Figure 6C). The ITGAV encoding integrin αV is the receptor for vitronectin, which is secreted by hepatocytes and abundant in the extracellular matrix (ECM) of liver,^[^ ^15^ ^]^ suggesting that integrin αV and vitronectin interaction may drive GC cell liver metastasis.

Further, visualization with IGV demonstrated that the abundance of KLF5 on the promoter of ITGAV was significantly decreased upon NAT10 knockout and increased upon NAT10 overexpression (Figure 6D), as subsequently confirmed by ChIP–qPCR (Figure 6E,F), and that the mRNA and protein levels of ITGAV were also tightly regulated by both KLF5 and NAT10 (Figure 6G–I and Figure S11A–E (Supporting Information)). Moreover, we observed that the protein level of ITGαV was increased upon NAT10 overexpression and was blocked by treatment with the NAT10 inhibitor Remodelin in GC organoids (Figure 6J). Notably, ITGAV expression was elevated in BGC823‐M3 cells compared to BGC823‐P cells (Figure S11F,G, Supporting Information). Additionally, IHC staining demonstrated that NAT10 overexpression resulted in dramatically increased ITGαV staining in the liver metastases of the model mice (Figure S11H, Supporting Information). Consistent with the findings from the mouse studies, ITGαV staining was also more abundant in the liver metastases of GC patients (Figure 6K,L and Figure S11I (Supporting Information)).

Furthermore, treatment of AGS cells with Cilengitide, an ITGαV inhibitor, led to a significant decrease in the cell adhesion ability (Figure S12A,B, Supporting Information). We confirmed that Cilengitide treatment suppressed the cell adhesion induced by NAT10 or KLF5 overexpression (Figure S12C–F, Supporting Information). We also found that knockdown of KLF5 and ITGαV using siRNAs could suppress the cell adhesion induced by NAT10 overexpression (Figure 6M,N). Collectively, these data indicate that NAT10‐mediated upregulation of KLF5 increases GC cell adhesion by activating the transcription of ITGαV.

### 2.7 Therapeutic Targeting of NAT10 and KLF5 Effectively Suppresses the Liver Metastasis of GC

**Figure 7:**
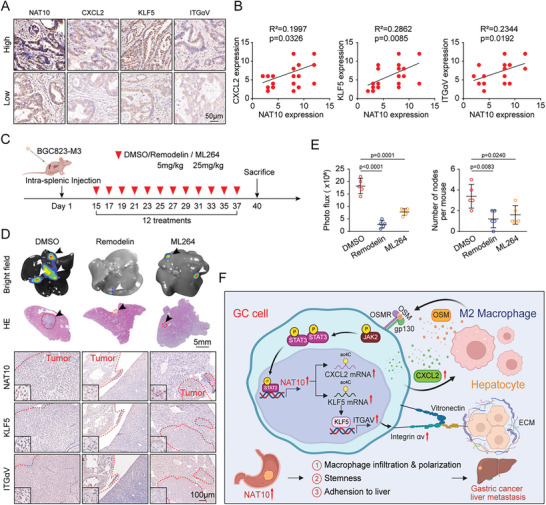
Therapeutic targeting of NAT10 and KLF5 effectively suppresses GCLM. A) The NAT10 expression level was significantly associated with the expression levels of CXCL2, KLF5, and ITGαV in 23 primary GC specimens from human patients with liver metastasis, as determined by IHC staining. B) NAT10 expression was positively correlated with CXCL2, KLF5, and ITGαV expression in GC specimens from patients with liver metastasis (linear regression). C–E) Therapeutic targeting of NAT10 and KLF5 inhibited the liver metastasis of BGC‐M3 cells injected intrasplenically into nude mice (*n* = 5). (C) Schematic overview of the experimental design showing the intrasplenic injection of nude mice with BGC‐M3 cells followed by treatment of the mice with Remodelin and ML264. (D) Representative images of the metastatic nodules in the liver (upper panel, bright field; middle panel, H&E staining (scale bars = 5 mm); lower panel, IHC staining of NAT10, KLF5, and ITGαV (scale bars = 100 µm)) and (E) quantification of the bioluminescence intensity and number of metastatic nodules. Arrow: liver metastasis. F) Model of the mechanism proposed in this study. GC, gastric cancer; IHC, immunohistochemical. Data are represented as mean ± SEM of three independent experiments. NS, not significant.

To explore the clinical significance of the NAT10/CXCL2/KLF5/ITGαV axis in liver metastasis, we first examined the expression of NAT10, CXCL2, KLF5, and ITGαV in the primary tumor tissues of GC patients with liver metastasis (cohort 3), and the results revealed that NAT10 expression was positively correlated with CXCL2, KLF5, and ITGαV expression in the 23 GC patients with liver metastasis (*R* ^2^ = 0.1997, *p* = 0.0326; *R* ^2^ = 0.2862, *p* = 0.0085; and *R* ^2^ = 0.2344, *p* = 0.0192, respectively). Similarly, the expression of KLF5 was also positively correlated with that of ITGαV (*R* ^2^ = 0.2838, *p* = 0.0089) (**Figure** **7**A,B).

Upon recognizing the crucial role of the NAT10/CXCL2/KLF5/ITGαV axis in GCLM, in vivo experiments were conducted, and the results revealed that the inhibitors significantly suppressed the liver metastasis of BGC‐M3 cells, as shown by bioluminescence imaging and the reduced number and size of liver metastatic lesions (Figure 7C–E). Furthermore, hematoxylin and eosin (H&E) staining revealed no significant toxicity to major organs, including the heart, liver, lungs, and kidneys (Figure S13A, Supporting Information). Overall, these findings suggest that targeting the NAT10/CXCL2/KLF5/ITGαV axis may be a promising approach for effectively restraining the liver metastasis of GC.

## 3 Discussion

Liver metastasis is a life‐threatening condition for GC patients, with no effective clinical treatments currently available. It is imperative to investigate the mechanism driving the metastasis of GC cells to the liver. In this study, we were surprised to find that the ac4C level is significantly increased in GC with liver metastasis, mainly due to the upregulation of the acetyltransferase NAT10. Mechanistically, NAT10‐mediated ac4C modification of CXCL2 mRNA can increase CXCL2 mRNA stability. Secreted CXCL2 promotes the infiltration and polarization of M2‐like macrophages to produce oncostatin M, which transcriptionally activates NAT10 expression via STAT3 signaling, establishing a positive feedback loop that contributes to GCLM. In addition, NAT10 mediated the ac4C modification of KLF5 mRNA and increases its stability. Then, KLF5 functions as a transcription factor that activates ITGαV expression, facilitating GC cell attachment to liver tissues. Notably, inhibition of the STAT3/NAT10/KLF5/ITGαV axis effectively suppressed liver metastasis in GC, suggesting that this axis could serve as a promising predictive biomarker and therapeutic target for GC with liver metastasis (Figure 7F).

Numerous studies have highlighted the critical role of TAMs in the TME, where they contribute to tumor development and immunotherapy resistance by creating an immunosuppressive microenvironment. TAMs abolish local immune surveillance by directly suppressing T‐cell activities through the secretion of immunosuppressive factors such as arginase 1 and IL10.^[^ ^16^ ^]^ Recent studies have revealed that TAM‐derived CCL20 promotes CCR6^+^ Treg infiltration, potentially contributing to resistance to anti‐PD‐L1 therapy in hepatocellular carcinoma.^[^ ^6d^ ^]^ Recent studies have also shown that various biological processes in immune cells, including macrophages, are influenced by RNA modifications. For example, IGF2BP2 can act as an m6A reader to maintain TSC1 and PPARG mRNA expression, influencing the repolarization of M1‐like macrophages to M2‐like macrophages through the TSC1–mTORC1 pathway and PPAR‐γ‐mediated fatty acid uptake.^[^ ^17^ ^]^ In our study, scRNA‐seq analysis revealed a gradual increase in the macrophage proportion in metastatic tissues. Furthermore, we found that NAT10 plays a crucial role in promoting the recruitment and polarization of M2‐like macrophages by increasing the stability of CXCL2 mRNA in an ac4C‐dependent manner in liver metastatic GC cells. Moreover, M2‐like macrophages can maintain the transcriptional activation of NAT10 in GC cells via the OSM/JAK2–STAT3 axis, establishing a positive feedback loop that contributes to GCLM.

RNA modifications, especially ac4C, and its corresponding acetyltransferase, NAT10, have gained attention in various cancers, including GC.^[^ ^10^, ^11^ ^]^ For example, it has been reported that NAT10‐mediated ac4C modification could promote GC progression by enhancing COL5A1 mRNA stability.^[^ ^10c^ ^]^ Another study revealed that ac4C modification of NOTCH3 mRNA is a key regulatory switch for its function in esophageal squamous cell carcinoma metastasis.^[^ ^10b^ ^]^ However, whether NAT10 and ac4C modification can regulate the progression of liver metastasis in patients with GC is still unknown. Here, analysis of our acRIP‐seq and RNA‐seq data identified KLF5 as the key transcription factor regulated by NAT10. KLF5 is generally regarded as a stemness regulatory factor for tumor cells.^[^ ^14a^ ^]^ Here, our study is the first to show that KLF5 facilitates the adhesion of GC cells to the ECM and hepatocytes by promoting the transcription of ITGαV, which contributes to the selective metastatic spread of GC cells to the liver. Integrin αV is reported to bind with vitronectin, a glycoprotein, which is abundant in retina pigment epithelium, serum, and especially hepatocytes, to regulate cell adhesion, tissue remodeling, tumor metastasis, and immunity.^[^ ^18^ ^]^ Integrins have been extensively studied in various cancers and have been identified as potential therapeutic targets.^[^ ^19^ ^]^ The expression of integrins is closely associated with specific cancer types and their tendency to metastasize to specific organs; for example, previous research has highlighted the involvement of integrin αvβ5 in GC and liver metastasis.^[^ ^19a,b^ ^]^ Our data also reveal that NAT10‐mediated ac4C modification of KLF5 increases GC cell adhesion by activating the transcription of ITGαV, which specifically contributes to GCLM.

Importantly, the expression of NAT10 was positively correlated with that of CXCL2, KLF5, and ITGαV in GC tissues, highlighting the clinical significance of the NAT10/CXCL2/KLF5/ITGαV axis in promoting liver metastasis. Encouragingly, targeting NAT10 and KLF5 with their specific inhibitors led to a significant decrease in the liver metastasis of GC cells. These findings suggest that NAT10 and KLF5 could be potential therapeutic targets and may be useful for patient stratification in clinical practice.

In summary, the findings presented in this study demonstrate a significant correlation between NAT10 and liver metastasis in GC. This finding suggests that NAT10 could serve as a valuable prognostic marker for GC. Additionally, the restoration of NAT10/KLF5 expression may constitute a novel therapeutic approach for GC patients with liver metastasis.

## 4 Experimental Section

### scRNA‐seq and Data Analysis

Two endoscopic biopsies of primary GC tissues and ultrasound‐guided puncture biopsies of liver metastatic tissues were collected for scRNA‐seq analysis. Briefly, fresh mucosal samples were dissected using iris scissors and digested in a phosphate‐buffered saline (PBS)–collagen II/IV solution for 30 min at 37 °C with agitation at 800 rpm. Following digestion, a 45 µm filter was used to filter the cell suspension. Centrifugation at 500 rpm for 6 min at 4 °C was performed to remove dead cells and red blood cells. The cells were then washed, resuspended in PBS supplemented with 0.5% fetal bovine serum (FBS), and loaded into microfluidic channels. A droplet‐based sequencing platform (10x Genomics) was used to generate a cDNA library. Library preparation was performed with the Single Cell 3′ Library Gel Bead Kit V2 (10x Genomics), and the library was subsequently sequenced on an Illumina NovaSeq 6000 system, aiming for a minimum of 100 000 150‐bp paired‐end reads per cell.

Data obtained via 10x Genomics sequencing were aligned and quantified against a human reference genome (hg19) using Cell Ranger software (version 3.1). The Seurat R package (version 3.2) was used to process the sparse gene expression matrices. Genes present in at least three cells and cells with feature counts of 500 to 6000 were retained, while those with >50% mitochondrial RNA reads were excluded. Data normalization (NormalizeData function), data scaling (ScaleData function, default settings), and identification of highly variable genes were performed using the FindVariableFeatures function. Specifically, Harmony (RunHarmony function) was run with the default settings using the 29 best fit PCs, resulting in corrected PC embeddings; this step was followed by shared nearest neighbor finding (FindNeighbors function, dims = 1:29), clustering (FindClusters function, resolution = 0.5), and uniform manifold approximation and projection (UMAP) dimensionality reduction (RunUMAP function, dims = 1:29). For detailed cell annotation, a list of marker genes was obtained from a public database and previous work.^[^ ^20^ ^]^ Differential gene expression analysis was conducted utilizing the FindAllMarkers function (multiple‐condition comparisons) or FindMarkers function (two‐condition comparisons), and genes with adjusted *p* < 0.05 and average logarithmic fold change ≥ 1 were considered significantly differentially expressed. Prediction of normal/tumor status without tumor annotation was performed by CopyKAT (https://github.com/navinlabcode/copykat) with the standard parameters. Aneuploid cells were classified as tumor cells, while diploid cells were classified as normal cells. GSVA and gene set enrichment analysis were conducted on selected differentially expressed genes to investigate the potential biological functions and pathways distinguishing primary tumor clusters from liver metastasis clusters.

To determine the activity of TFs in specific GC subpopulations, the SCENIC+ program in R was utilized to reconstruct gene‐regulatory networks and pinpoint stable cell states based on cis‐regulatory cues using default parameters. Initially, TF–gene coexpression modules were established using GENIE3 via a data‐centric approach. These modules were subsequently refined through analysis with the RcisTarget package to enrich for genes associated with the corresponding TF binding motifs. Upon obtaining the regulons, the AUCell activity was assessed for each regulon across individual cells, resulting in a binary regulon activity matrix.^[^ ^21^ ^]^

### Patients and Specimens

Cohorts of GC patients who underwent radical gastrectomy without adjuvant radiotherapy or chemotherapy at Nanjing Drum Tower Hospital, the Affiliated Hospital of Nanjing University Medical School (Nanjing, Jiangsu, China), were included. GC cohort 1 comprised 75 patients, cohort 2 comprised 192 patients, and cohort 3 comprised 23 patients who underwent radical gastrectomy between January 14, 2014, and January 4, 2016. Paired cancerous GC and normal gastric mucosal tissue specimens were embedded in paraffin to construct a TMA, and clinicopathological features, including age, sex, and TNM stage (American Joint Committee on Cancer classification), were recorded. Additionally, fresh‐frozen pathologically confirmed cancerous GC and normal gastric mucosal tissue and normal liver tissue specimens obtained from recent patients at Nanjing Drum Tower Hospital were included. The normal tissue was obtained at a minimum of 3 cm distance from the tumor, and normal histology was assessed by routine examination. These tissues were obtained for GC organoid culture after written informed consent was provided. This study was approved by the Institutional Review Boards of Nanjing Drum Tower Hospital and the First Affiliated Hospital of Anhui Medical University.

### Cell Lines and Cell Culture

The AGS GC cell line was purchased from the American Type Culture Collection (MD, USA), and the BGC823 and MGC803 GC cell lines, THP‐1 cell line, and HBE cell line were obtained from the Type Culture Collection of the Chinese Academy of Sciences (Shanghai, China). MKN45 cells were obtained from the cell bank of the RIKEN BioResource Center (Tsukuba, Japan). AGS cells were cultured in F12K medium (Cellcook Biotech Co., Ltd., Guangzhou, China), BGC823, THP‐1, and MKN45 cells were cultured in RPMI‐1640 medium (Invitrogen Life Technologies, CA, USA), and MGC803 and HBE cells were cultured in Dulbecco's modified Eagle medium (Invitrogen Life Technologies, CA, USA). The culture media were supplemented with 10% FBS (Wisent, Montreal, Canada), 100 µg mL^−1^ streptomycin, and 100 U mL^−1^ penicillin (New Cell & Molecular Biotech, Suzhou, China), and all cells were cultured in an incubator with 5% CO_2_ at 37 °C. The cells were stored at −80 °C using CELLSAVING (New Cell & Molecular Biotech, Suzhou, China). All cells tested negative for mycoplasma contamination and were authenticated based on short tandem repeat profiling before use. All the inhibitors, cytokines, and chemokines used in this study are listed in Table S1 (Supporting Information).

### Isolation of Primary Hepatocytes

Normal human liver tissue was minced, cut into 20–50 small pieces, and normal mouse liver tissue was perfused in situ with 50 mL of Gibco liver perfusion medium (Invitrogen Life Technologies, CA, USA) followed by 45 mL of Gibco liver digestion medium (Invitrogen Life Technologies, CA, USA). The liver digests were filtered through a cell strainer and washed with Gey's balanced salt solution (Sigma, USA) containing DNase I (2 mg mL^−1^, Sigma, USA). The homogenate was subjected to 3 rounds of centrifugation at 50 × *g* for 2 min at room temperature. Hepatocytes were collected from the cell pellets and washed thoroughly with PBS.

### IHC Staining and TMA Analysis

As described in the previous study,^[^ ^9a^ ^]^ standard protocols were used for IHC staining. Staining of NAT10, CXCL2, p‐STAT3 (Y705), KLF5, and ITGαV in the TMA was independently scored by two pathologists blinded to the clinical data by applying a semiquantitative immunoreactivity score (IRS) in the cohort, as reported previously.^[^ ^9a^ ^]^ Under these conditions, samples with an IRS of 0–6 and samples with an IRS of 8–12 were considered to have low and high expression, respectively, of NAT10, CXCL2, KLF5, and ITGαV. The antibodies used are listed in Table S2 (Supporting Information).

### IF Assay

The details of the IF assay had been described previously.^[^ ^9b^ ^]^ Briefly, GC cells were fixed with paraformaldehyde and incubated first with an anti‐NAT10 antibody at 4 °C overnight and then with the corresponding Alexa‐Fluor‐conjugated secondary antibody (Beyotime, Shanghai, China) at a 1:500 dilution for another hour at room temperature. Next, the cells were incubated with DAPI (Beyotime, Shanghai, China) for 5 min. The protocols used for IF staining of ac4C in the TMA were similar to those used for IHC staining, except that the tissues were incubated with the indicated primary antibody at 4 °C overnight and then with the corresponding Alexa‐Fluor‐conjugated secondary antibody, as described for the cellular IF assay mentioned above. Images of cells and TMA tissues were acquired with a Leica DMi8 system. The antibodies used are listed in Table S2 (Supporting Information).

### MIF Staining

Multiplex immunofluorescence staining was performed using a PANO 5‐plex immunohistochemistry kit (Cat# 10080100100, Panovue) according to the manufacturer's instructions as described previously.^[^ ^20b^ ^]^ Briefly, tissues from patients and mice with liver metastasis were formalin‐fixed, paraffin‐embedded, and sectioned. The sections were deparaffinized, rehydrated, and subjected to antigen retrieval. The sections were then blocked for 10 min with 10% bovine serum in PBS and incubated with specific primary antibodies overnight. After washing, the sections were incubated with the corresponding secondary antibody for 1 h at room temperature, and amide signal amplification was performed. Images of the tissues were acquired with a Leica DMi8 system. The antibodies used are listed in Table S2 (Supporting Information).

### siRNA, shRNA, sgRNA, Plasmid Transfection, and Lentiviral Transduction

siRNAs targeting NAT10 and KLF5 were designed and synthesized by RiboBio (Guangzhou, China). shRNAs targeting NAT10 and STAT3 were designed based on siRNA sequences and were then subcloned and inserted into pLVX vectors (pLVX‐puro), which were constructed by YouBio (Changsha, China). The sequences are listed in Table S3 (Supporting Information). Plasmids encoding the STAT3‐CA and STAT3‐DN mutants were obtained from Addgene (Plasmid #24983, Plasmid #24984). NAT10‐OE lentiviruses were constructed by GeneChem Co., Ltd. (Shanghai, China) using GV341 vectors (Ubi‐MCS‐3FLAG‐SV40‐puromycin). NAT10‐OE lentiviruses expressing the G641E or K290A mutant were produced according to the methods used for production of the wild‐type NAT10‐OE lentivirus by Corues Biotechnology (Nanjing, China). KLF5‐OE lentiviruses were produced by Corues Biotechnology (Nanjing, China) using pLv10ltr vectors (PGK‐CMV‐3HA‐SV40‐hygromycin). siRNAs were transfected into cells with DharmaFECT4 (Dharmacon, Chicago, IL, USA). All of the plasmids were transfected into cells with Lipofectamine 3000 (Invitrogen, Grand Island, NY, USA). shRNA lentiviruses or overexpression lentiviruses and their corresponding vector control lentiviruses were incubated with GC cells. Approximately 12 h later, the lentivirus‐containing medium was removed, and new culture medium was added. After 72 h, infected GC cells were selected with 1 µg mL^−1^ puromycin or 1 mg mL^−1^ hygromycin (Sigma, USA). To generate AGS NAT10‐KO and KLF5‐KO cells, the NAT10 sgRNAs and KLF5 sgRNAs listed in Table S3 (Supporting Information) were designed and were then cloned and inserted into a plasmid coexpressing a sgRNA and Cas9 (pCas‐puro‐U6‐KO) by Corues Biotechnology (Nanjing, China), and NAT10‐KO and KLF5‐KO cells were generated as previously reported.^[^ ^9a^ ^]^

### Organoid Culture

The organoid culture method was described in the previous study.^[^ ^9a^ ^]^ Briefly, approximately GC tissue samples with a volume of ≈1 cm^3^ obtained from different GC patients were minced, washed with 1× chelating buffer (5.6 mm Na_2_HPO_4_, 8.0 mm KH_2_PO_4_, 96.2 mm NaCl, 1.6 mm KCl, 43.4 mm sucrose, 54.9 mm d‐sorbitol, and 0.5 mm dl‐dithiothreitol (pH = 7)), and cut into 20–50 small pieces. These GC tissues were digested, and glands from the tissues were seeded in basement matrix. Five hundred microliters of medium containing growth factors (50 ng mL^−1^ EGF, 100 ng mL^−1^ noggin, 10% R‐spondin1, 50% Wnt‐conditioned medium, 200 ng mL^−1^ FGF10, 1 nm gastrin, 2 µm TGF‐beta inhibitor and 10 µm RHOKi) was added to each well. Once organoids formed, the NAT10 expression level in different organoids and transfected suitable organoids was measured with the NAT10‐overexpressing or the corresponding control lentiviral vector or they were with the NAT10 inhibitor Remodelin for 24 h. The organoids were then fixed and subjected to H&E staining, and the expression of NAT10, KLF5, and ITGαV was evaluated by staining as described previously.^[^ ^9^, ^20^ ^]^ The organoid culture procedure was first approved by the Institutional Review Board of Nanjing Drum Tower Hospital and the First Affiliated Hospital of Anhui Medical University.

Gastric organoids derived from the stomachs of TFF1^∆/∆^ mice were established as human GC organoids and cultured in the same medium. The organoids were then digested into single cells, and NAT10 sgRNA (cloned and inserted into a plasmid coexpressing the sgRNA and Cas9 (pCas‐puro‐U6‐KO)) was transfected into the cells. TFF1^∆/∆^‐NAT10‐KO organoids were selected using puromycin (1 µg mL^−1^).

### Animal Studies

BALB/c male nude mice (5–6 weeks old) were purchased from Nanjing Biomedical Research Institute of Nanjing University (Nanjing, Jiangsu, China) and bred under specific pathogen‐free conditions in sterile ventilated racks in the animal care facility at the Anhui Medical University. The study was approved by the Animal Care and Use Committee of the Anhui Medical University. First, BGC823‐GFP‐luc cells (1 × 10^6^, named BGC‐P cells) were injected into nude mice through the splenic vein, and metastatic cells were extracted from the liver for reinjection. The mouse liver metastasis model was established by 3 rounds of the above procedure, and the metastatic cells isolated in rounds 1, 2, and 3 were named BGC‐M1, BGC‐M2, and BGC‐M3, respectively.

BGC823‐luc cells (1 × 10^6^) stably overexpressing NAT10 and the corresponding control cells were injected into the splenic vein of nude mice (BGC‐CON, *n* = 10; BGC‐NAT10, *n* = 9). Liver metastasis was examined weekly by bioluminescence imaging, and the detailed experimental procedures had been described previously.^[^ ^9a^ ^]^ The mice were sacrificed 2 months later, and the livers were then fixed in 4% paraformaldehyde for further analyses. Furthermore, NAT10‐deficient MKN45 cells (1 × 10^6^) and the corresponding control cells were injected intrasplenically into nude mice (*n* = 5 mice per group).

For the orthotopic model, a TFF1^∆/∆^‐NAT10‐KO organoid suspension (2 × 10^5^ cells per mouse) was directly injected into the submucosa of the lower third of the stomach, and the mice were sacrificed 2 months later. In addition, BGC‐M3 cells (1 ×1 0^6^) were injected into the splenic vein of nude mice (*n* = 5 mice per group), and the mice were then treated with inhibitors of NAT10 (Remodelin, 5 mg kg^−1^) and KLF5 (ML264, 25 mg kg^−1^) for a total of 12 treatments and sacrificed for further analyses.

### RNA‐seq

Total RNA was first extracted from the indicated GC cells for RNA‐seq, and the quality and quantity of the RNA were assessed with a NanoDropTM ND‐1000 spectrophotometer. Denaturing agarose gel electrophoresis was used to assess RNA integrity. mRNA extraction was performed using the NEBNextR Poly(A) mRNA Magnetic Isolation Module. RNA libraries were constructed using a KAPA Stranded RNA‐Seq Library Prep Kit (Illumina). Libraries were sequenced using the Illumina HiSeq 4000 platform. RNA‐seq was completed by Gene Denovo (Guangzhou, China).

### acRIP‐seq

For acRIP‐seq, more than 150 µg of purified total RNA was extracted, and the integrity and quantity of the RNA in each sample were assessed using agarose gel electrophoresis and a NanoDropTM instrument. Intact mRNA was first isolated from total RNA samples using an Arraystar Seq‐StarTM poly(A) mRNA Isolation Kit according to the manufacturer's protocol. The isolated mRNA was chemically sheared into 100‐nucleotide‐long fragments by incubation in fragmentation buffer (10 mm Zn^2+^ and 10 mm Tris‐HCl, pH 7.0), and the size of the mRNA fragments was confirmed via agarose gel electrophoresis. Then, the ac4C‐modified mRNAs were immunoprecipitated with an anti‐ac4C antibody (an aliquot of the fragmented mRNAs was used as input). The eluted ac4C mRNA fragments were then concentrated for RNA‐seq library construction. RNA‐seq libraries for the ac4C antibody‐enriched mRNAs and input mRNAs were prepared using a KAPA Stranded mRNA‐seq Kit (Illumina). The prepared libraries were diluted to a final concentration of 8 pm, and clusters were generated on an Illumina cBot system using a HiSeq 3000/4000 PE Cluster Kit (#PE‐410‐1001, Illumina) prior to sequencing on the Illumina HiSeq 4000 platform. For acRIP‐seq data analysis, the raw reads were trimmed by Trimmomatic software and aligned to the Ensembl reference genome by HISAT2 software (v2.1.0). The differential ac4C‐RIP‐enriched regions (peaks) between different pairs of groups were analyzed by exomePeak software. These differential peaks were annotated using the latest Ensembl database. Sequence motifs were one of the basic functional units of molecular evolution. The Multiple EM for Motif Elicitation and Discriminative Regular Expression Motif Elicitation algorithms were used to identify motifs among the ac4C peak sequences. acRIP‐seq was completed by Cloud‐seq (Shanghai, China). The antibodies used are listed in Table S2 (Supporting Information).

### qRT‐PCR

Total RNA was extracted from cells using TRIzol reagent (Invitrogen, CA, USA) according to the manufacturer's instructions. RT was performed with HiScript Q RT SuperMix for qPCR (Vazyme Biotech Co., Ltd., Nanjing, China). RT‐PCR was performed in triplicate with a SYBR Green PCR Kit (Vazyme Biotech Co., Ltd., Nanjing, China) on an Applied Biosystems 7900HT sequence detection system (Applied Biosystems). The primers used are listed in Table S4 (Supporting Information).

### Western Blotting

Western blotting was performed as previously described,^[^ ^22^ ^]^ and the antibodies used are listed in Table S2 (Supporting Information).

### Sphere Formation Assay

Sphere formation assays were performed as previously described.^[^ ^20b^ ^]^ Briefly, cells were seeded at a density of ≈2 cells µL^−1^ in ultralow attachment plates (Corning) in medium containing 2% B27, 20 ng mL^−1^ FGF10, and 20 ng mL^−1^ EGF. After 14 days, spheres larger than 50 µm in diameter were counted by light microscopy. Assays were carried out in triplicate.

### M2‐Like Macrophage Derived from THP‐1 Cell Recruitment Assay

GC cells were plated in 24‐well plates (Corning) at densities that allowed growth to 100% confluence within 24 h. The medium was then collected and added to the lower compartments of a 24‐well Transwell plate (Corning) with or without recombinant CXCL2 (3.6 nm; Novoprotein, Suzhou, China). THP‐1 cells were stimulated with PMA (100 ng mL^−1^) and polarized to the M2 phenotype using IL3 (20 ng mL^−1^) and IL14 (20 ng mL^−1^). M2‐like macrophages derived from THP‐1 cells were then seeded in the upper compartments (7 × 10^4^), and 16 h later, the migrated cells were fixed for 30 min in 4% paraformaldehyde, stained with crystal violet (Beyotime, Shanghai, China), and imaged.

### RNA Stability Assay

For the RNA stability assay, actinomycin D (2 µg mL^−1^; MCE, NJ, USA) was used to inhibit transcription. Total RNA was extracted from cells treated with actinomycin D for specific numbers of hours. The abundance of RNA remaining at each time point was normalized to the initial abundance (0 h).

### Enzyme‐Linked Immunosorbent Assay (ELISA)

The conditioned media of cultured cells were first lyophilized, and then the concentration of secreted CXCL2 was measured using CXCL2 ELISA kits (Ruixin Biotech, Quanzhou, China) following the manufacturer's instructions.

### Cell Adhesion Assay

To test the ability of GC cells to adhere to hepatocytes and respiratory cells, GC cells were then added to the culture media of hepatocytes and HBE cells for 4 h and washed with PBS 5 times, after which the adherent cells were imaged with a Leica DMi8 system. GC cells were also added to the culture medium of normal liver organoids for 6 h, after which images were captured.

### Dot Blot Assay

The dot blot assay was performed as previously reported.^[^ ^9a^ ^]^ Methylene blue (MB) was used to bind mRNA and as the loading control. The details of the antibodies used in this assay are listed in Table S2 (Supporting Information).

### RIP Assay

The RIP assay was performed as previously described.^[^ ^9a^ ^]^ Briefly, the MagnaRIP RNA‐Binding Protein Immunoprecipitation Kit (Millipore, MA, USA) was used according to the manufacturer's instructions. The corresponding cell lysates were incubated with beads coated with 5 µg of control IgG (Beyotime, Shanghai, China) or an anti‐ac4C antibody with rotation at 4 °C overnight. Then, total RNA was extracted for measurement of CXCL2 and KLF5 expression by qRT‐PCR. The antibodies used are listed in Table S2 (Supporting Information), and the primers used are listed in Table S4 (Supporting Information).

### ChIP Assay

The ChIP assay was performed using a ChIP assay kit (Beyotime, Shanghai, China). Briefly, GC cells were fixed with 1% formaldehyde for 10 min, the reaction was quenched with glycine at room temperature for 5 min, and the cells were washed, collected, and resuspended in lysis buffer. The cell lysates were subjected to ultrasonication to shear the genomic DNA. The sonicated chromatin solution was subjected to immunoprecipitation with anti‐STAT3 and anti‐KLF5 antibodies. Immunoprecipitated DNA was purified and analyzed by qRT‐PCR. The antibodies used are listed in Table S2 (Supporting Information), and the primers used are shown in Table S4 (Supporting Information).

### CUT&Tag Sequencing

To explore the downstream target genes regulated by KLF5, a CUT&Tag kit (Hyperactive Universal CUT&Tag Assay Kit for Illumina, TD903) was used according to the manufacturer's instructions. Sequence alignment, peak annotation, IGV visualization, and functional signaling pathway enrichment analysis were performed on the clean data. The antibodies used are listed in Table S2 (Supporting Information).

### Statistical Analysis

Statistical analyses were performed with SPSS 18.0 or GraphPad Prism 8 software. The significance of differences in the IRSs for NAT10/CXCL2/KLF5/ITGαV staining in primary tumor tissues was assessed by the Wilcoxon test (grouped data). Differences in OS were determined by the Kaplan–Meier method with the log‐rank test for significance. Univariate Cox regression analysis was used to estimate HRs and the associated 95% CIs. Then, the predictive value of the parameters was analyzed using receiver operating characteristic (ROC) curve analysis for censored data and the AUC of each ROC curve was calculated as previously reported.^[^ ^20b^ ^]^ Representative data were shown as the means ± standard deviations. A two‐tailed paired *t* test was used for within‐group comparisons, while a two‐sample *t* test was used for between‐group comparisons. One‐way ANOVA followed by Tukey's multiple comparisons test was used for comparisons among more than two groups, *p* < 0.05 was considered to indicate statistical significance, *p* ≥ 0.05 indicated a lack of significance, denoted NS. Experimental reports in the study were reliably reproduced in at least three independent experiments or by multiple biologically independent replicates.

### Data Availability Statement

The raw sequence data reported in this paper had been deposited in the Genome Sequence Archive (Genomics, Proteomics & Bioinformatics 2021) in National Genomics Data Center (Nucleic Acids Res 2021), China National Center for Bioinformation/Beijing Institute of Genomics, Chinese Academy of Sciences that were publicly accessible at https://ngdc.cncb.ac.cn/gsa.

- acRIP‐seq data and RNA‐seq data: Genome Sequence Archive HRA006162
- scRNA‐seq data: Genome Sequence Archive HRA006538

## Conflict of Interest

The authors declare no conflict of interest.

## Author Contributions

C.C., Z.W., and Q.L. contributed equally to this work. C.C., Z.W., and Q.L. performed the experiments; C.C. and L.B. contributed to the construction of organoids; M.L., L.X., S.Z., M.Z., Y.Q., and Q.D. evaluated the IHC of TMA; Y.F. was responsible for the pathological staining interpretation; X.Z., Z.M., J.X., and B.W. participated in the construction of plasmids; C.C., Z.W., M.W., and Q.W. analyzed data; C.C. and S.W. wrote the paper; S.W. designed the project.

## Supporting information



Supporting Information

## Data Availability

The data that support the findings of this study are available from the corresponding author upon reasonable request.
