# Supplementary material for: NAT10 Promotes Gastric Cancer Liver Metastasis by Modulation of M2 Macrophage Polarization and Metastatic Tumor Cell Hepatic Adhesion
Source: Adv Sci (Weinh). 2025 Feb 22;12(15):2410263. doi: 10.1002/advs.202410263 (PMC12005778; doi:10.1002/advs.202410263)
Supplement: Supplementary file 1 — Supporting Information [file ADVS-12-2410263-s001.docx]

**Supporting Information**

**NAT10 promotes gastric cancer liver metastasis by** **modulation of M2 macrophage polarization and metastatic tumor cell hepatic adhesion**

Chen Chen^1,8^, Zhangding Wang^1,8^, Qingfeng Lin^2,8^, Mengmeng Li^3^, Lei Xu^4^, Yao Fu^5^, Xiaoya Zhao^3^, Zhuang Ma^3^, Jiawen Xu^3^, Shimeng Zhou^3^, Mingyue Zhang^3^, Yun Qian^3^, Linsen Bao^6^, Bo Wang^1^, Meng Wang^6^, Qingqing Ding^7^, Qiang Wang^1*^, Shouyu Wang^1,3*^

**This PDF file includes:**

**Table S1.** Inhibitors, cytokines or chemokines.

**Table S2.** Antibodies for Western blot (WB), RIP-qPCR, acRIP-qPCR, IF, Dot blot (DB), CUT&Tag and IHC.

**Table S3.** The sequences of siRNAs and sgRNAs.

**Table S4.** The Oligonucleotides used in this study.

**Figure S1.** sc-RNAseq and data analyses.

**Figure S2.** Generation of cells with stable NAT10 overexpression and knockout.

**Figure S3.** NAT10 recruits and polarizes M2-like macrophages via N4-acetylcytidine modification of CXCL2.

**Figure S4.** M2-like macrophage-secreted OSM activates NAT10 transcription in liver metastatic GC via STAT3 signaling.

**Figure S5.** NAT10 increases the adhesion of GC cells to hepatocytes.

**Figure S6.** Visualization of candidate genes with IGV.

**Figure S7.** KLF5 expression is positively regulated by NAT10 in liver metastatic GC.

**Figure S8.** NAT10 promotes the stemness of GC cells.

**Figure S9.** KLF5 promotes the adhesion of GC cells.

**Figure S10.** Reciprocal modulation of KLF5 expression reverses the changes in the adhesion and stemness of GC cells caused by alterations in NAT10 expression.

**Figure S11.** ITGαV expression is positively regulated by the NAT10/KLF5 axis.

**Figure S12.** NAT10- and KLF5-induced adhesion of GC cells was blocked by Cilengitide.

**Figure S13.** Remodelin and ML264 have no obvious systemic toxicity.

**Supporting Tables**

**Table S1 Inhibitors, cytokines or chemokines.**

| **chemicals** | **Source** | **Identifier** |
| --- | --- | --- |
| rCXCL2 | Novoprotein | Cat# C096 |
| Stattic | Sigma-Aldrich | Cat# 573099 |
| PMA | Sigma-Aldrich | Cat# P8139 |
| LPS | Beyotime | Cat# S1732 |
| IFN γ | Peprotech | Cat# 300-02 |
| IL4 | Peprotech | Cat# 200-04 |
| IL13 | Peprotech | Cat# 200-13 |
| OSM | Peprotech | Cat# 300-10-10 |
| EGF | Peprotech | Cat# 315-09-100 |
| Noggin | Peprotech | Cat# 250-38-100 |
| FGF10 | Peprotech | Cat# 100-26-25 |
| Gastrin | Sigma-Aldrich | Cat# G9145 |
| TGF-beta inhibitor | Tocris | Cat# 2939 |
| RHOKi | MCE | Cat# HY-10071 |
| B27 | Thermo | Cat# 7504044 |
| Actinomycin D | MCE | Cat# HY-13918 |
| Remodelin | MCE | Cat# HY-16706 |
| ML264 | Selleck | Cat# S8196 |
| Cilengitide | Selleck | Cat# S6387 |
| DharmaFECT4 | Dharmacon | Cat# T-2004-03 |
| Lipofectamine 3000 | Invitrogen | Cat# L3000015 |

**Table S2 Antibodies for Western blot (WB), RIP-qPCR, acRIP-qPCR, IF, Dot blot (DB), CUT&Tag and IHC.**

| **Antibodies** | **Source** | **Identifier** |
| --- | --- | --- |
| anti-m7G (for DB) | MBL | Cat# RN017M |
| anti-m6A (for DB) | Abcam | Cat# ab284130 |
| anti-m5C (for DB) | MBL | Cat# D346-3 |
| anti-m1A (for DB) | MBL | Cat# D345-3 |
| anti-ac4C (for DB, IF, and RIP-qPCR) | Abcam | Cat# ab253039 |
| anti-CD68 (for IF) | Proteintech | Cat# 28058-1-AP |
| anti-CD206 (for IF) | CST | Cat# 24595 |
| anti-NAT10 (for WB, IHC, and IF) | Abcam | Cat# ab194297 |
| anti-CXCL2 (for WB, IHC) | Proteintech | Cat# 26791-1-AP |
| anti-KLF5 (for WB, IHC, CHIP-qPCR and CUT&Tag) | Proteintech | Cat# 21017-1-AP |
| anti- STAT3 (for CHIP-qPCR) | CST | Cat# 9139 |
| anti-p-STAT3(Y705) (for WB, IHC) | CST | Cat# 9145S |
| anti-ITGαV (for WB and IHC) | ABcolonal | Cat# A2091 |
| anti-Rabbit IgG (for RIP-qPCR,CUT&Tag and CHIP-qPCR) | Beyotime | Cat# A7016 |
| anti-HRP mouse (for WB) | Beyotime | Cat# A0216 |
| anti-HRP rabbit (for WB) | Beyotime | Cat# A0208 |
| anti-IHC rabbit (for IHC) | Servicebio | Cat# G1215 |
| anti-IHC mouse (for IHC) | Servicebio | Cat# G1216 |
| anti-β-Actin (for WB) | Beyotime | Cat# AF0003 |
| anti-GAPDH (for WB) | Beyotime | Cat# AF0006 |

**Table S3 The sequences of siRNAs and sgRNAs.**

| **siRNAs** | **Sequences (5’-3’)** |
| --- | --- |
| NAT10 siRNA#1 | GTACTCCAATATCTTTGTT |
| NAT10 siRNA#2 | GGATGTGCATTCCAGGTAC |
| KLF5 siRNA#1 | GGACACTCTTAATGTTTCT |
| KLF5 siRNA#2 | GATGTGAAATGGAGAAGTA |
| STAT3 siRNA | CCCGGAAAUUUAACAUUCU |
| ITGAV siRNA | CGACAGGCTCACATTCTACTT |
| **sgRNAs** | **Sequences (5’-3’)** |
| NAT10 sgRNA#1 | CTCCCACGTTGCCACCATGG |
| NAT10 sgRNA#2 | GGAGTTGAAGGAGAGCTTGC |
| NAT10 sgRNA#3 | CTGCTGTAAGACTCTAGACC |
| KLF5 sgRNA#1 | ACGGTCTCTGGGATTTGTAG |
| KLF5 sgRNA#2 | GAGGGGCAGTCGTTTCACTC |
| KLF5 sgRNA#3 | TCACCTCTGGAGCTGCGGTC |

**Table S4 The Oligonucleotides used in this study.**

| **Names** | **Primer sequences for qRT-PCR (5’-3’)** |
| --- | --- |
| NAT10 F**^a^** | GGGATTGGCCTGCAGCATA |
| NAT10 R**^b^** | GGCTCCATGACCACATCCTT |
| GAPDH F | CATGTGGGCCATGAGGTCCACCAC |
| GAPDH R | GGGAAGCTCACTGGCATGGCCTTCC |
| CD163 F | TTTGTCAACTTGAGTCCCTTCAC |
| CD163 R | TCCCGCTACACTTGTTTTCAC |
| CD206 F | GGGTTGCTATCACTCTCTATGC |
| CD206 R | TTTCTTGTCTGTTGCCGTAGTT |
| CXCL2 F | CGCACAGCCGCTCGAA |
| CXCL2 R | CCATTCTTGAGTGTGGCTATGACT |
| CXCL3 F | CCAAACCGAAGTCATAGCCAC |
| CXCL3 R | TGCTCCCCTTGTTCAGTATCT |
| CXCL8 F | GGAGAAGTTTTTGAAGAGGGCTG |
| CXCL8 R | ACAGACCCACACAATACATGAAG |
| OSM F | GAGCAGCTGACAAGGTCTGG |
| OSM R | TCAGCCGTGTCTGAGTTGTC |
| IL6 F | CCACCGGGAACGAAAGAGAA |
| IL6 R | GAGAAGGCAACTGGACCGAA |
| IL11 F | GAGTTTCCCCAGACCCTCGG |
| IL11 R | GTAGGACAGTAGGTCCGCTC |
| IL27 F | AGTTCACAGTCAGCCTGCAT |
| IL27 R | GCAGGTGAGATTCCGCAAAG |
| CNTF F | TTCACAGAGCATTCACCGCT |
| CNTF R | TGGGGTAAAATGCACCTGCT |
| LIF F | CCAACGTGACGGACTTCCC |
| LIF R | TACACGACTATGCGGTACAGC |
| CTF1 F | CCCTCCTCGTCTGCATGGTA |
| CTF1 R | GAGGCCAAAGGGAACTGAGG |
| KLF5 F | ACACCAGACCGCAGCTCCA |
| KLF5 R | TCCATTGCTGCTGTCTGATTTGTAG |
| ITGAV F | GACAGTCCTGCCGAGTA |
| ITGAV R | CTGGGTGGTGTTTGCT |
| AMMECR F | TCTGCTCAGTGTCTCTGCTCA |
| AMMECR R | CGGTGCGTTTTGATCCTTTTTCA |
| CRLF3 F | GAAAGTGCATCACAGACAAGGG |
| CRLF3 R | TCTGGCAGTCATCTAGTGGTTT |
| DDIT4 F | TGAGGATGAACACTTGTGTGC |
| DDIT4 R | CCAACTGGCTAGGCATCAGC |
| DEPDC1 F | TTTTGGTCCTGAAGTTACAAGGC |
| DEPDC1 R | TGGATACCTTCGTGGTAGAGTTT |
| FOSL2 F | CAGAAATTCCGGGTAGATATGCC |
| FOSL2 R | GGTATGGGTTGGACATGGAGG |
| GJB3 F | TACTGAGCGGTGTGAACAAGT |
| GJB3 R | GCATGTGACGAAGATGAGCTG |
| GPT2 F | GTGATGGCACTATGCACCTAC |
| GPT2 R | TTCACGGATGCAGTTGACACC |
| GRK5 F | CCAACACGGTCTTGCTGAAAG |
| GRK5 R | TCTCTGTCTATGGTCCTTCGG |
| LRCH1 F | ACTCTGCACCCACTTCATCAT |
| LRCH1 R | GGTACGGGGAAATTCCTTCAAT |
| MAFF F | GGTGGATCTGGTAACAAAGTCTG |
| MAFF R | CGCTCAGCTCTCGCTTGAT |
| NET1 F | GAGCCAAGCAATAAAAGAGTTCG |
| NET1 R | TGGGACTGTTGACCTGCTAGA |
| NPIPA7 F | CTGGCACTCTGCTTGGGTTA |
| NPIPA7 R | GTGCAGAGGGAGATGGCAAA |
| PDLIM5 F | TCAACATGCCTCTGACAATCTC |
| PDLIM5 R | GCCTTGCCGCCATCTTTTAG |
| PHLDB2 F | TGGTGCATTCTGTTGAGAACG |
| PHLDB2 R | CAGGCACAGGTTGTGAGAG |
| PTK2B F | CCCCTGAGTCGAGTAAAGTTGG |
| PTK2B R | GATACGCACGTCCTCCTTTTC |
| WEE1 F | AGGGAATTTGATGTGCGACAG |
| WEE1 R | CTTCAAGCTCATAATCACTGGCT |
| ZNF48 F | TGCCCGGATCAAACACCAG |
| ZNF48 R | GGGACCGAGGAATCTTTGGG |
| **Names** | **Primer sequences for acRIP-qPCR (5’-3’)** |
| CXCL2 F | CGCCAGTATTTCTGACCAAC |
| CXCL2 R | CCAGTGCTTGCAGACCCT |
| KLF5 F | AGGTACGTGCGCTCGCGGTTCT |
| KLF5 R | TCCGGGCGGCCACTTTCTCC |
| **Names** | **Primer sequences for CHIP-qPCR (5’-3’)** |
| NAT10 F | TTCAGACATTTGCGGTAT |
| NAT10 R | TTTATTGGTTTCGCTTGG |
| ITGAV BS1 F | GTAGATTGGGACGCTGGACC |
| ITGAV R1 | GCTGAGCACCTGAGGAGGC |
| ITGAV F2 | CTGCACTCACGGAAGTACGCT |
| ITGAV R2 | GCACTGGGAGGAGACCTGGAA |

a: F, Forward; b: R, Reverse.

**Supporting Figures and Figure Legends:**

**
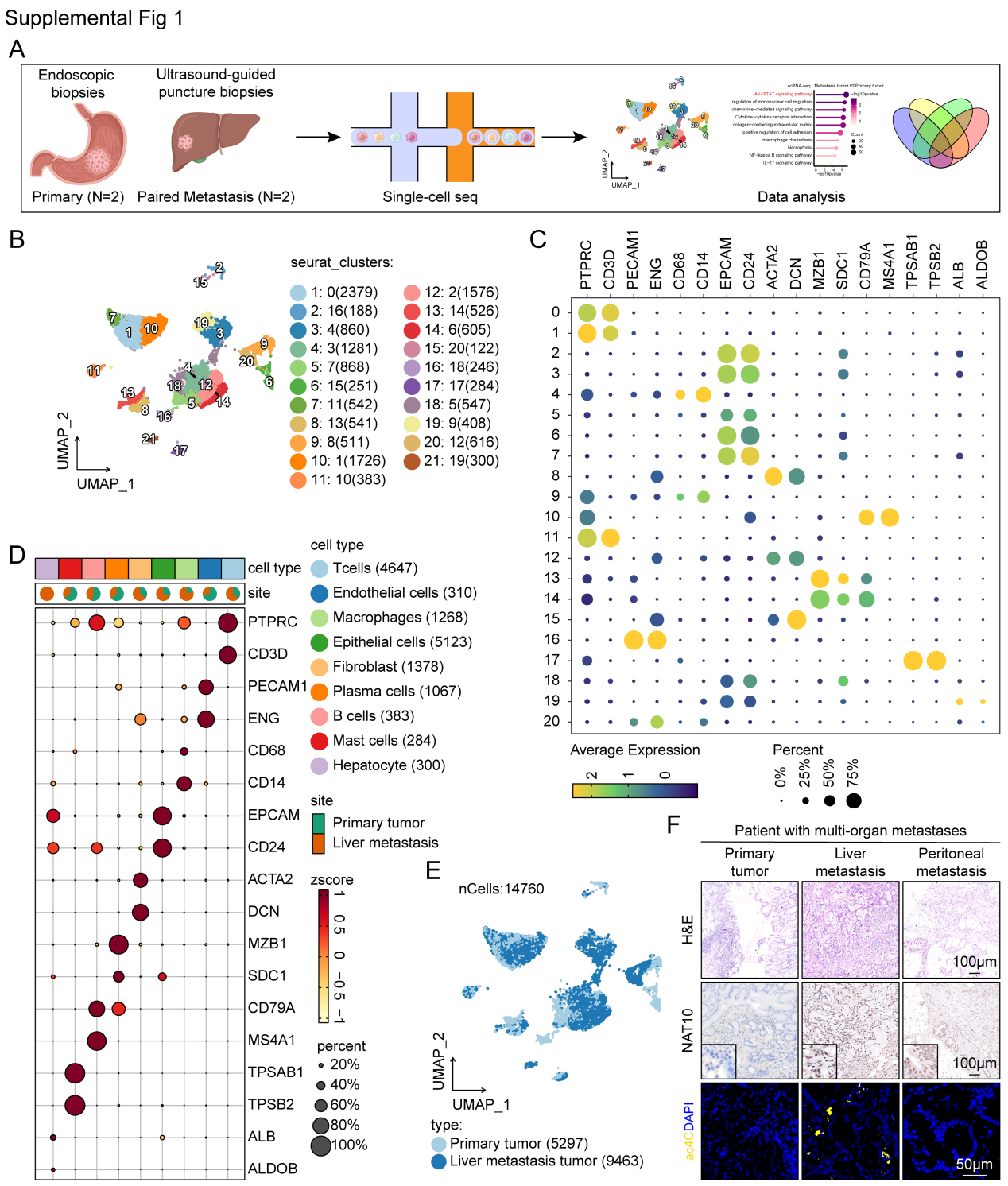
**

**Figure S1 sc-RNAseq and data analyses.**

**A**, Schematic diagram of the workflow for the whole scRNA-seq study. Two endoscopic biopsies of primary GC tissue and two ultrasound-guided puncture biopsies of paired liver metastatic tissues were included. **B**, UMAP analysis representative of the 21 clusters generated from the total population of high-quality cells. **C**, Dot plot of representative canonical marker genes mapped to each cell cluster. **D**, Dot plot of representative canonical marker genes mapped to each cell type. **E**, UMAP plot showing different sample origins of the total cell population. **F**, H&E staining (scale bars=100 µm), IHC staining of NAT10 (scale bars=100 µm) and IF staining of ac4C (scale bars=50 µm) in the primary tumor, liver metastasis, and peritoneal metastasis of one patient with multiorgan metastases. GC, gastric cancer; UMAP, uniform manifold approximation and projection.


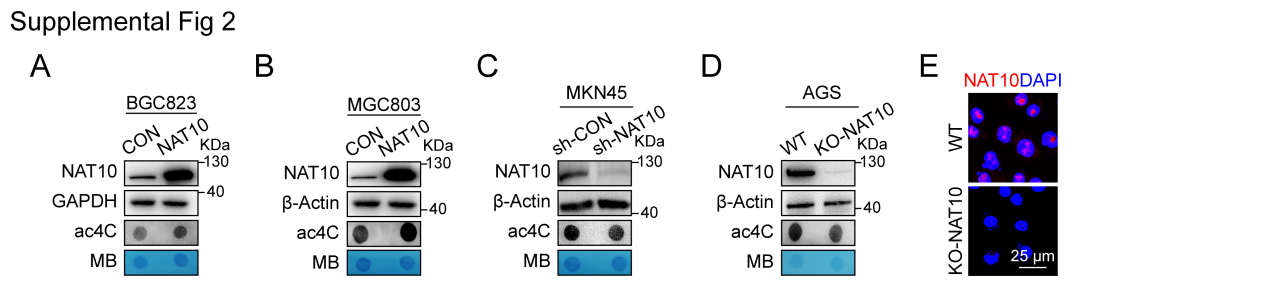


**Figure S2 Generation of cells with stable NAT10 overexpression and knockout.**

**A-B**, The overexpression efficiency of NAT10 was verified at the translational level in BGC823 and MGC803 cells by western blotting. **C-D**, The knockdown and knockout efficiency of NAT10 was verified at the translational level in MKN45 and AGS cells by western blotting. **E**, IF staining of NAT10 in AGS-KO cells (scale bars=25 µm). IF, immunofluorescence; KO, knockout. The data are presented as the means ± SDs of three independent experiments.


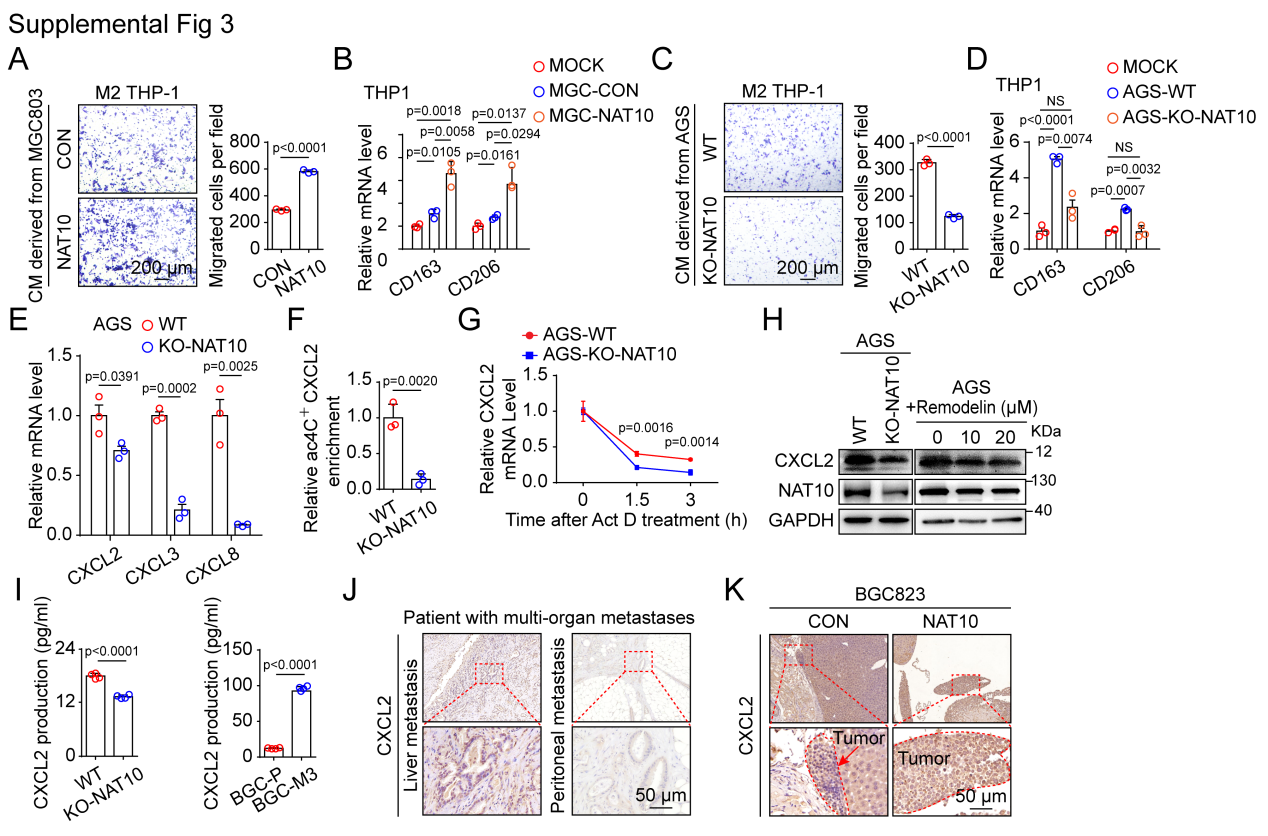


**Figure S3 NAT10 recruits and polarizes M2-like macrophages via N4-acetylcytidine modification of CXCL2.**

**A-B**, NAT10 promoted the recruitment of macrophages. (**A**, **C**) M2-like macrophages derived from THP-1 cells that migrated through uncoated filter membranes after 16 h of culture in medium derived from MGC803 cells with NAT10 overexpression, AGS cells with NAT10 knockout and the corresponding control cells. The migrated cells were stained with crystal violet, visualized via microscopy (left panel), and counted (right panel) (scale bar=200 μm). (**B**, **D**) q-PCR was conducted to measure the mRNA levels of M2-like markers (CD163 and CD206) in THP-1 cells cultured with medium derived from MGC803 cells overexpressing NAT10, AGS cells with NAT10 knockout and the corresponding control cells. **E**, The mRNA levels of CXCL2, CXCL3 and CXCL8 in NAT10-KO GC cells were measured by qRT‒PCR. **F**, acRIP-qPCR analysis was employed to demonstrate NAT10-mediated CXCL2 ac4C modification. ac4C modification of CXCL2 decreased upon knockout of NAT10. **G**, The level of CXCL2 expression in NAT10-KO and the corresponding control GC cells treated with actinomycin D (2 µg/mL) at the indicated time points were measured by qRT‒PCR. **H**, The protein level of CXCL2 in NAT10-KO and Remodelin-treated AGS cells was measured by western blotting. **I**, The concentration of CXCL2 in the culture supernatant of NAT10-KO AGS cells and BGC-M3 cells was measured by ELISA. **J**, The protein level of CXCL2 in liver metastatic and peritoneal metastatic tumor tissues of a GC patient with multiorgan metastases was evaluated by IHC staining (scale bars=50 µm). **K**, Sections of liver metastases formed from intrasplenically injected NAT10-OE BGC823 cells in nude mice were subjected to IHC staining with an anti-CXCL2 antibody (scale bars=50 µm). GC, gastric cancer; KO, knockout; OE, overexpressing. The data are presented as the means ± SDs of three independent experiments. NS, not significant.


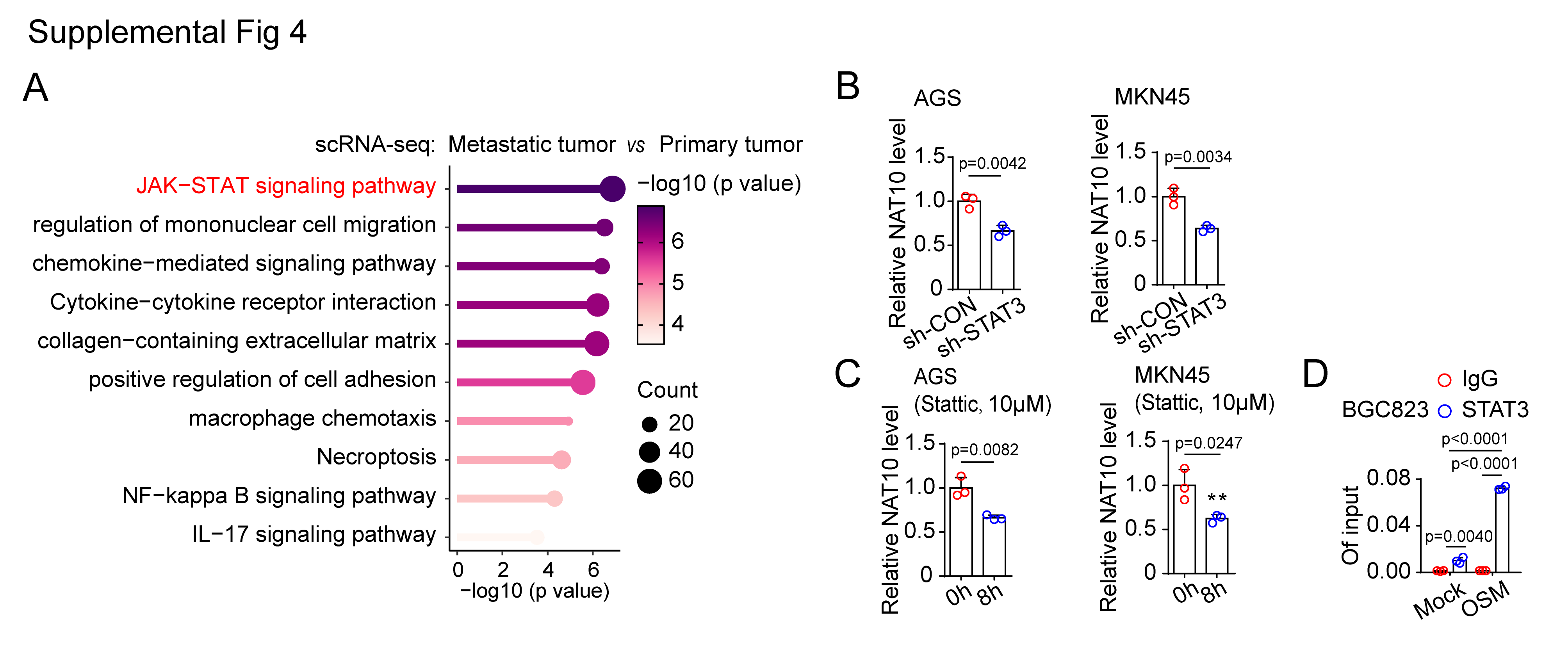


**Figure S4 M2-like macrophage-secreted OSM activates NAT10 transcription in liver metastatic GC via STAT3 signaling.**

**A**, KEGG enrichment analysis results showing that the JAK-STAT signaling pathway was enriched in genes expressed in metastatic tumor cells. **B**-**C**, The mRNA level of NAT10 in (**B**) STAT3-KD AGS cells, STAT3-KD MKN45 cells and (**C**) AGS cells and MKN45 cells treated with a STAT3 inhibitor (Stattic, 10 μM) was measured by qRT‒PCR. **D**, Enrichment of STAT3 at the NAT10 promoter, as determined by ChIP‒qPCR, upon OSM (50 ng/ml) treatment. KEGG, Kyoto Encyclopedia of Genes and Genomes; KD, knockdown. The data are presented as the means ± SDs of three independent experiments. NS, not significant.


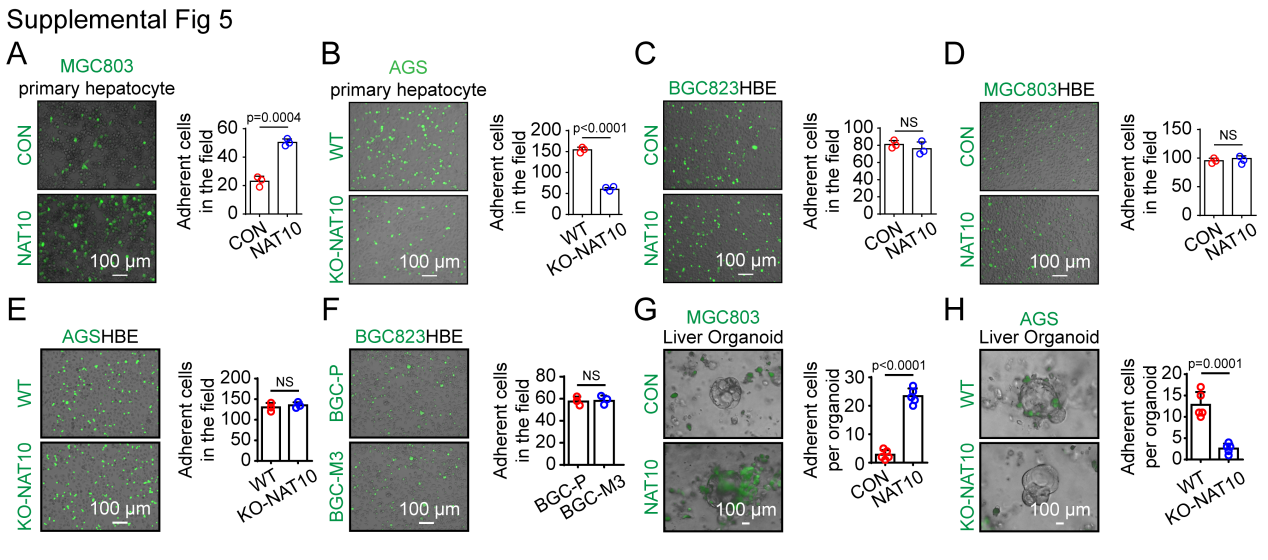


**Figure S5 NAT10 increases the** **adhesion of GC cells to hepatocytes.**

**A**-**H**, NAT10 significantly increased the adhesion of GC cells to hepatocytes. (**A**-**B**) Representative images of NAT10-OE MGC803 cells and NAT10-KO AGS cells adhering to hepatocytes (left panel, scale bars=100 μm) and quantification of the adherent cells (right panel). (**C**-**F**) Representative images of NAT10-OE BGC823 cells, NAT10-OE MGC803 cells, NAT10-KO AGS cells and BGC-M3 cells adhering to HBE cells (left panel, scale bars=100 μm) and quantification of the adherent cells (right panel). (**G**-**H**) Representative images of NAT10-OE MGC803 cells and NAT10-KO AGS cells adhering to liver organoids (left panel, scale bars=100 μm) and quantification of the adherent cells (right panel). OE, overexpressing; KO, knockout. The data are presented as the means ± SDs of three independent experiments. NS, not significant.


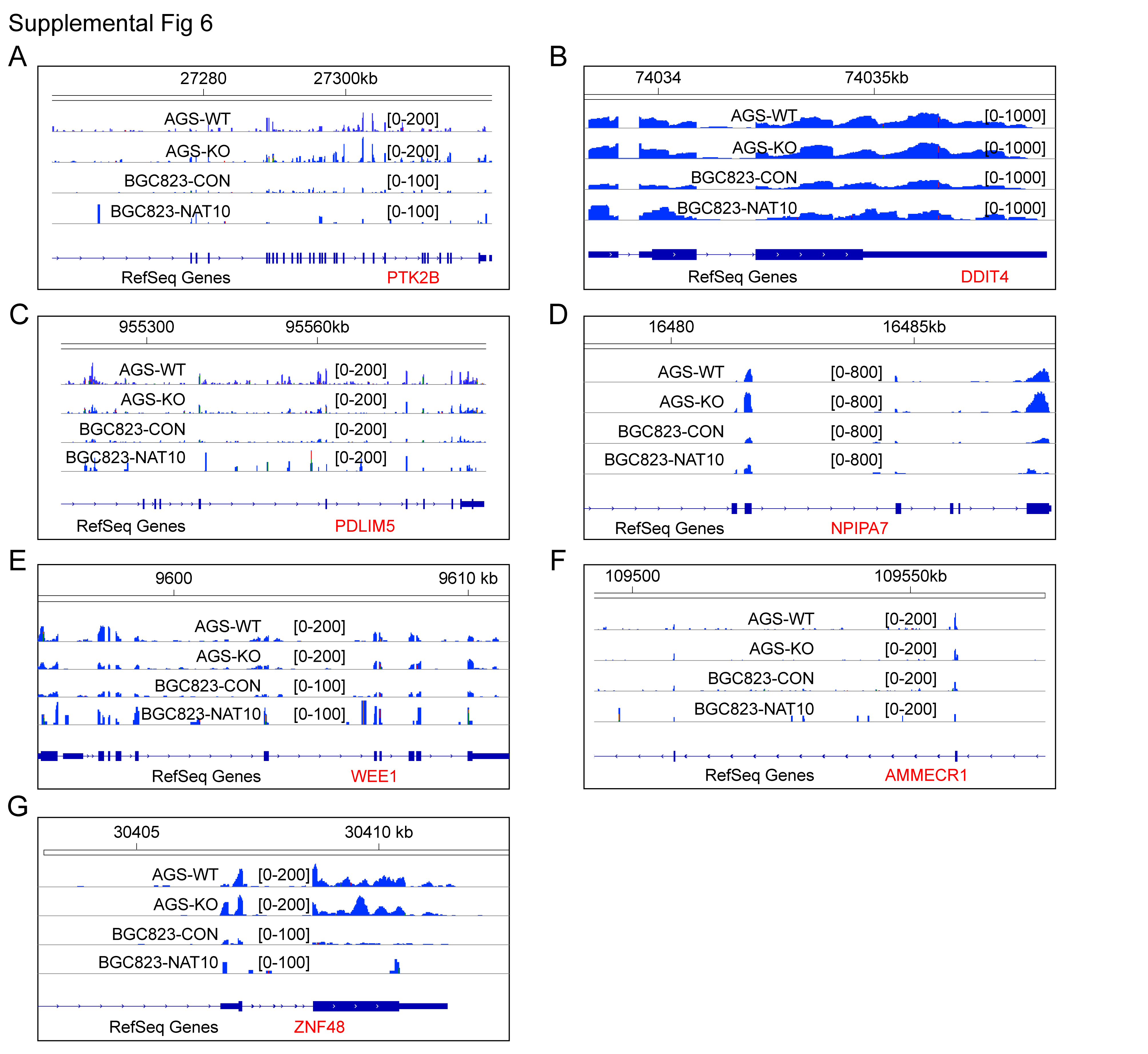


**Figure S6 Visualization of candidate genes with IGV.**

**A**-**G**, The ac4C abundances on (**A**) PTK2B, (**B**) DDIT4, (**C**) PDLIM5, (**D**) NPIPA7, (**E**) WEE1, (**F**) AMMECR1, and (**G**) ZNF48 mRNA transcripts in BGC823 cells and AGS cells, as determined by acRIP-seq.

IGV, Integrative Genomics Viewer.


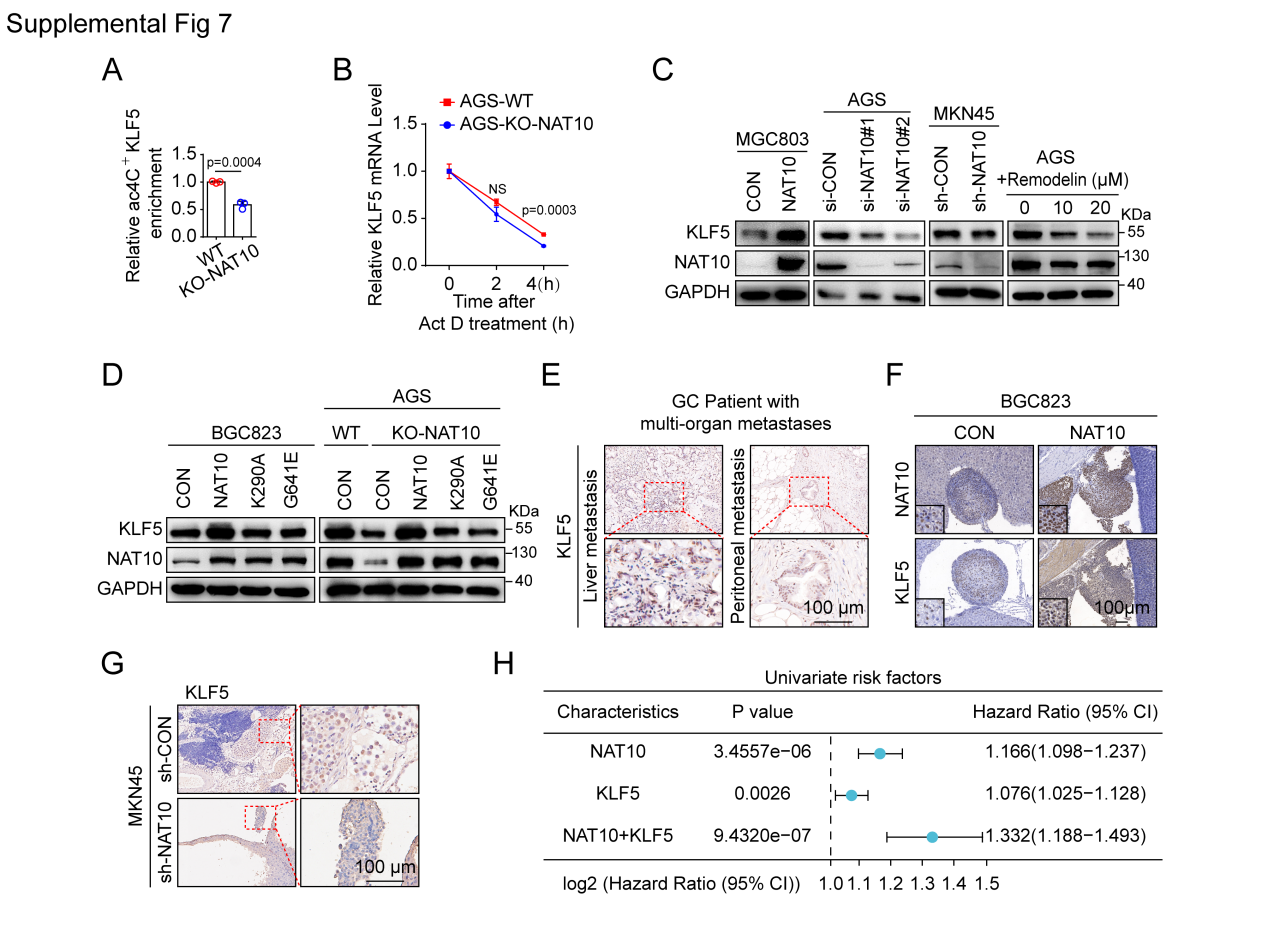


**Figure S7 KLF5 expression is positively regulated by NAT10 in liver metastatic GC.**

**A**, acRIP-qPCR analysis was employed to demonstrate NAT10-mediated KLF5 ac4C modification. ac4C modification of KLF5 decreased upon knockout of NAT10. **B**, The level of KLF5 expression in NAT10-KO and the corresponding control GC cells treated with actinomycin D (2 µg/mL) at the indicated time points was measured by qRT‒PCR. **C**, The protein level of KLF5 in NAT10-OE MGC803 cells, NAT10-KD AGS cells, NAT10-KD MKN45 cells and Remodelin-treated AGS cells was measured by western blotting. **D**, The protein level of KLF5 in BGC823 cells overexpressing wild-type NAT10 or NAT10 mutants (lacking a functional acetyltransferase domain (G641E) or RNA helicase domain (K290A)) and in NAT10-KO AGS cells was measured by western blotting. **E**, The protein level of KLF5 in liver metastatic and peritoneal metastatic tumor tissues of a GC patient with multiorgan metastases was evaluated by IHC staining (scale bars=100 µm). **F**-**G**, Sections of liver metastases derived from (**F**) NAT10-OE BGC823 cells and (**G**) NAT10-KD MKN45 cells intrasplenically injected into nude mice were subjected to IHC staining for KLF5 (scale bars=100 µm). **H**, Univariate analyses were performed in GC cohort 2. GC, gastric cancer; OE, overexpressing; KO, knockout; KD, knockdown; CA, constitutively active. The data are presented as the means ± SDs of three independent experiments. NS, not significant.


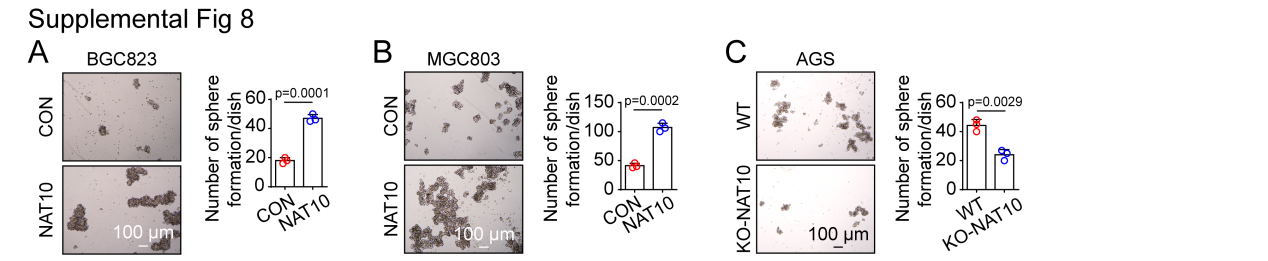


**Figure S8 NAT10 promotes the stemness of GC cells.**

**A**-**C**, Representative images of sphere formation assays in (**A**) NAT10-OE BGC823 cells, (**B**) NAT10-OE MGC803 cells and (**C**) NAT10-KO AGS cells (left panel, scale bars=100 μm) and quantification of sphere formation (right panel).

OE, overexpressing; KO, knockout. The data are presented as the means ± SDs of three independent experiments. NS, not significant.


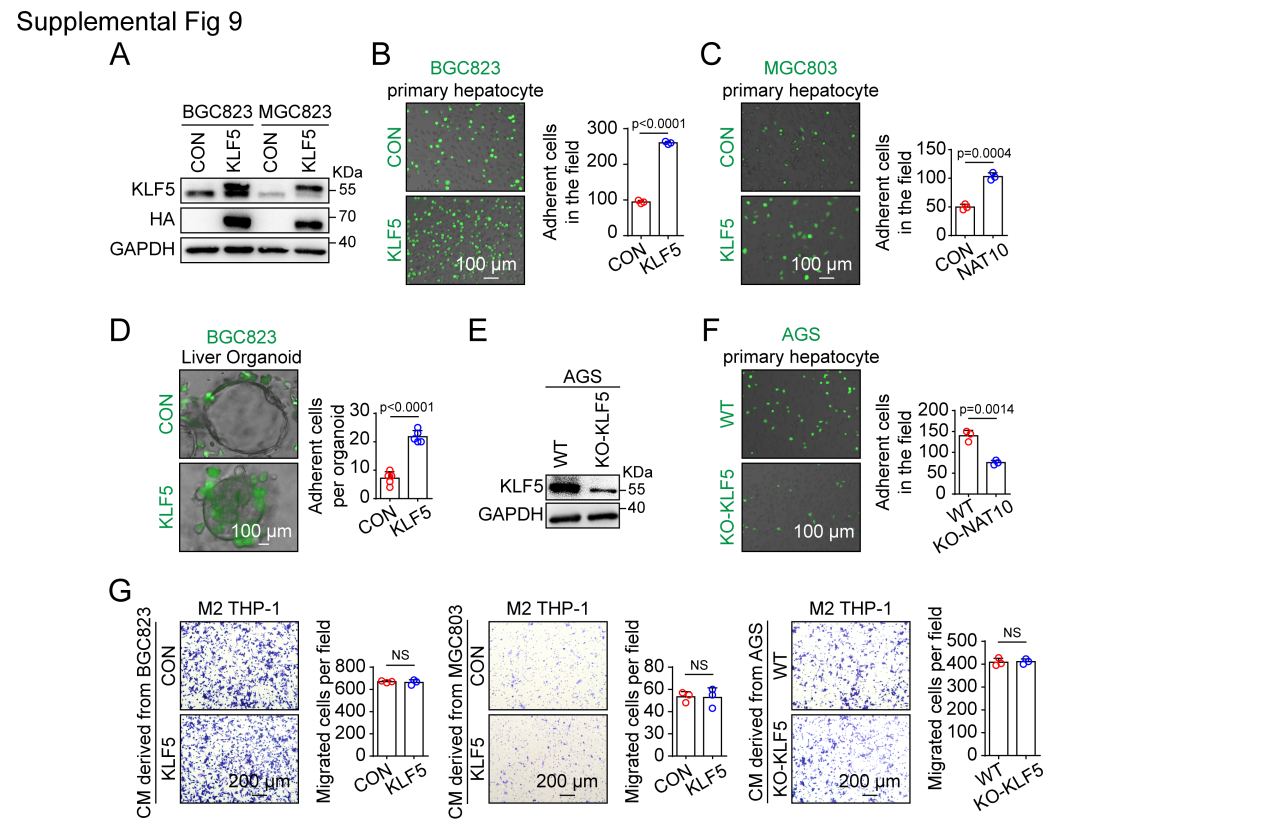


**Figure S9 KLF5 promotes the adhesion of GC cells.**

**A**, The overexpression efficiency of KLF5 was verified at the translational level in BGC823 and MGC803 cells by western blotting. **B**-**D**, KLF5 significantly promoted the adhesion of GC cells. Representative images of (**B**) KLF5-OE BGC823 cells and (**C**) KLF5-OE MGC803 cells adhering to hepatocytes (left panel, scale bars=100 μm) and quantification of the adherent cells (right panel). (**D**) Representative images of NAT10-OE BGC823 cells adhering to normal human liver organoids (left panel, scale bars=100 μm) and quantification of the adherent cells (right panel). **E**, The knockout efficiency of KLF5 was verified at the translational level in AGS cells by western blotting. **F**, Representative images of KLF5-KO AGS cells adhering to hepatocytes (left panel, scale bars=100 μm) and quantification of the adherent cells (right panel). **G**, M2-like macrophages derived from THP-1 cells that migrated through uncoated filter membranes after 16 h of culture in medium derived from KLF5-OE BGC823 cells, KLF5-OE MGC803 cells or KLF5-KO AGS cells. The cells were stained with crystal violet, visualized via microscopy (left panel, scale bar=200 μm), and counted (right panel). GC, gastric cancer; OE, overexpressing; KO, knockout. The data are presented as the means ± SDs of three independent experiments. NS, not significant.

**
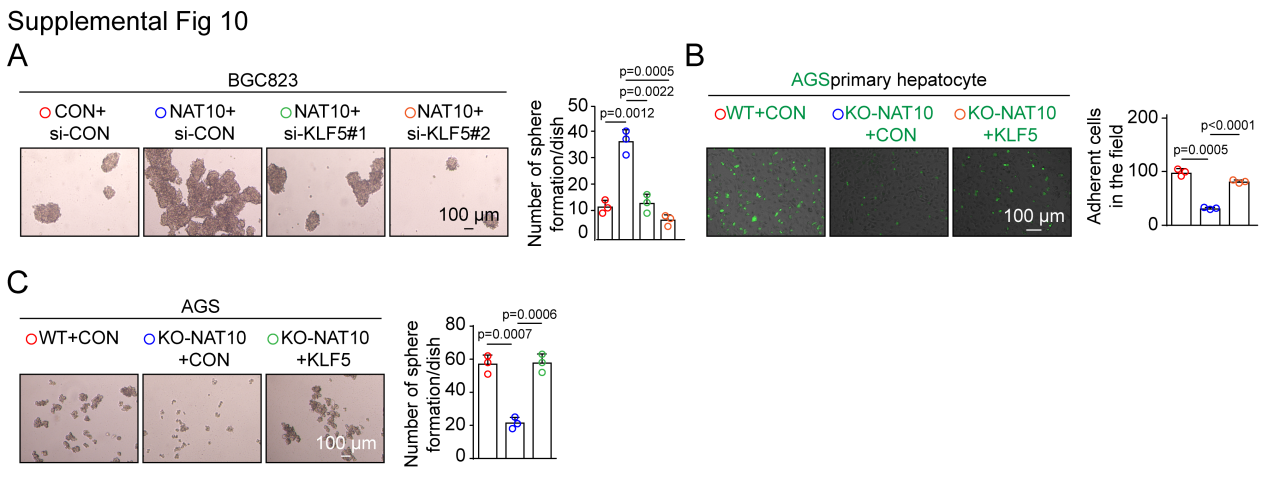
**

**Figure S10 Reciprocal modulation of KLF5 expression reverses the changes in the adhesion and stemness of GC cells caused by alterations in NAT10 expression.**

**A**, Representative images (left panel, scale bars=100 µm) and quantification (right panel) of sphere formation by NAT10-OE BGC823 cells transfected with si-KLF5. **B**, Representative images of NAT10-KO AGS cells transfected with the KLF5 overexpression plasmid adhering to hepatocytes (left panel, scale bars=100 μm) and quantification of the adherent cells (right panel). **C**, Representative images of sphere formation by KLF5-transfected NAT10-KO AGS cells (left panel, scale bars=100 μm) and quantification of sphere formation (right panel). OE, overexpressing; KO, knockout. The data are presented as the means ± SDs of three independent experiments. NS, not significant.


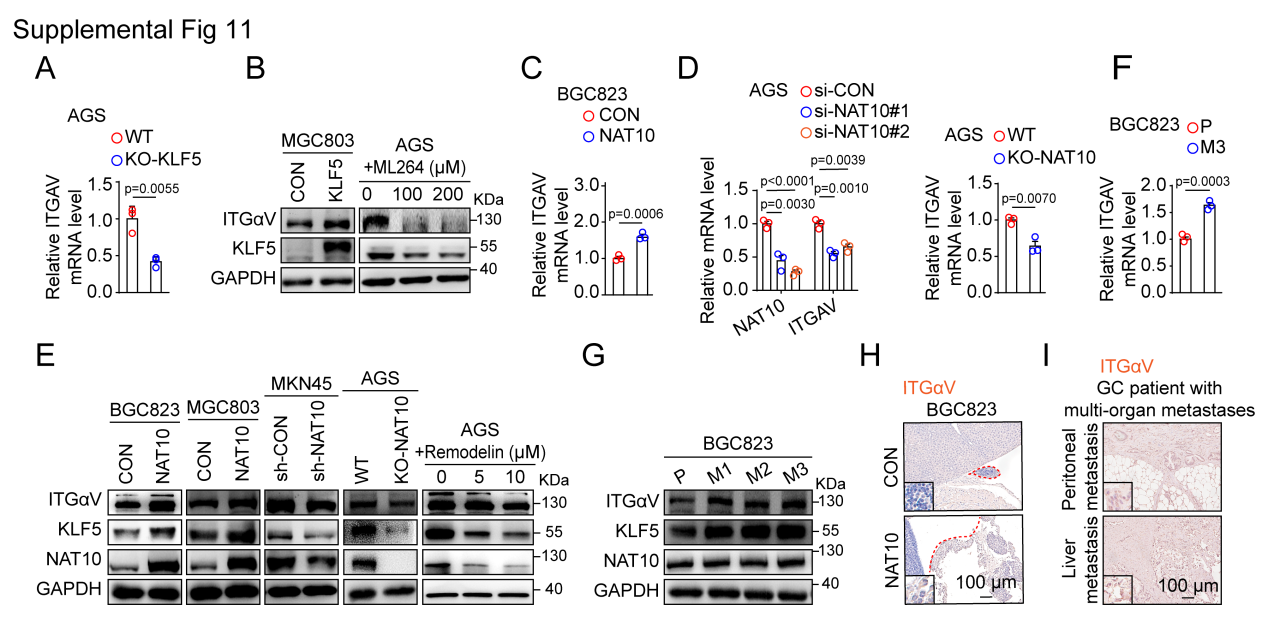


**Figure S11 ITGαV expression is positively regulated by the NAT10/KLF5 axis.**

1. **B**, The mRNA and protein levels of ITGAV (encoding ITGαV) in KLF5-OE MGC803 cells, KLF5-KO AGS cells and AGS cells treated with ML264 were measured by qRT‒PCR and western blotting. **C**-**E**, The mRNA and protein levels of ITGAV (encoding ITGαV) in NAT10-OE BGC823 cells, NAT10-OE MGC803 cells, NAT10-KO AGS cells, AGS cells transfected with si-NAT10, NAT10-KD MKN45 cells and AGS cells treated with Remodelin were measured by qRT‒PCR and western blotting. **F**-**G**, The mRNA and protein of ITGAV (encoding ITGαV) in BGC-M3 cells were measured by qRT‒PCR and western blotting. **H**, Sections of liver metastases derived from intrasplenically injected NAT10-OE BGC823 cells in nude mice were subjected to IHC staining with an anti-ITGαV antibody (scale bars=100 µm). **I**, The protein level of ITGαV in liver metastatic and peritoneal metastatic tumor tissues from a GC patient with multiorgan metastases was evaluated by IHC staining (scale bars=100 µm). GC, gastric cancer; OE, overexpressing; KO, knockout; KD, knockdown. The data are presented as the means ± SDs of three independent experiments. NS, not significant.

**
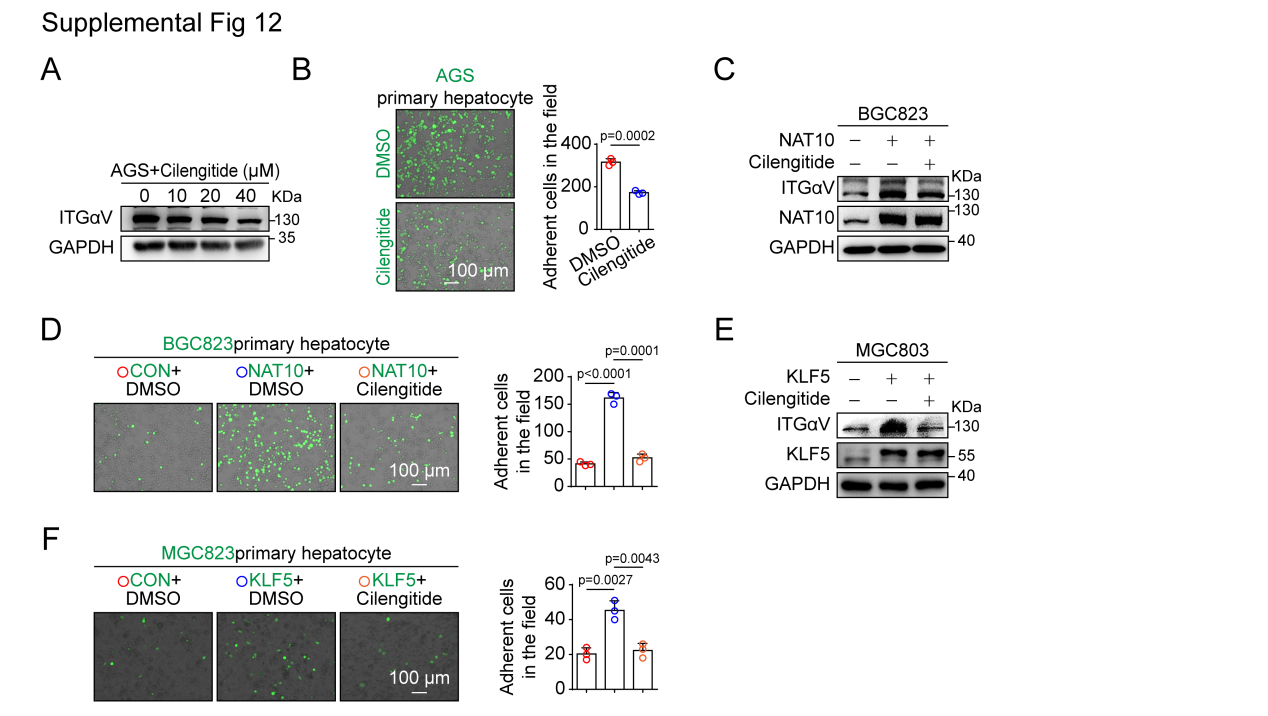
**

**Figure S12 NAT10- and KLF5-induced adhesion of GC cells was blocked by Cilengitide.**

**A**, The protein expression of ITGαV in AGS cells treated with Cilengitide was measured by western blotting. **B**, Representative images of AGS cells treated with Cilengitide adhering to hepatocytes (left panel, scale bars=100 μm) and quantification of the adherent cells (right panel). **C**-**D**, Cilengitide treatment suppressed the increase in cell adhesion caused by NAT10 overexpression. (**C**) The protein expression level of ITGαV was measured in NAT10-OE BGC823 cells treated with Cilengitide by western blotting. (**D**) Representative images (left panel, scale bars=100 µm) and quantification (right panel) of NAT10-OE BGC823 cells treated with Cilengitide (40 µM) that adhered to hepatocytes. **E**-**F**, Cilengitide treatment suppressed the increase in cell adhesion caused by KLF5 overexpression. (**E**) The protein expression level of ITGαV was measured in KLF5-OE MGC803 cells treated with Cilengitide by western blotting. (**F**) Representative images (left panel, scale bars=100 µm) and quantification (right panel) of KLF5-OE MGC803 cells treated with Cilengitide (40 µM) that adhered to hepatocytes.GC, gastric cancer; OE, overexpressing; KO, knockout. The data are presented as the means ± SDs of three independent experiments. NS, not significant.


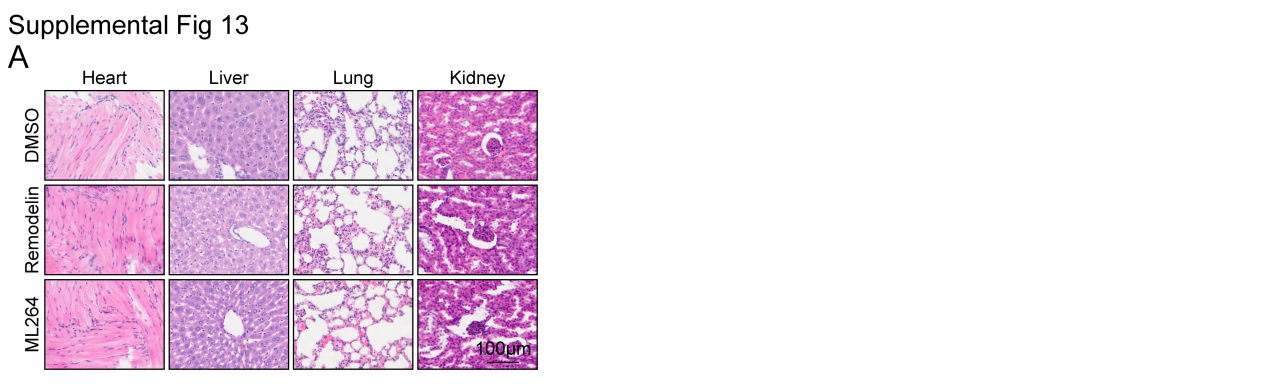


**Figure S13 Remodelin and ML264 have no obvious systemic toxicity.**

**A**, Representative H&E staining of major organs, including the heart, liver, lungs and kidneys, harvested from mice treated with or without Remodelin and ML264 at the end of the experiment (scale bars=100 μm).
